# Systemic and Mucosal Immune Responses to Sublingual or Intramuscular Human Papilloma Virus Antigens in Healthy Female Volunteers

**DOI:** 10.1371/journal.pone.0033736

**Published:** 2012-03-16

**Authors:** Zhiming Huo, Sara L. Bissett, Raphaela Giemza, Simon Beddows, Clarissa Oeser, David J. M. Lewis

**Affiliations:** 1 Infectious Diseases, St George's - University of London, London, United Kingdom; 2 Virus Reference Department, Health Protection Agency, London, United Kingdom; 3 Surrey Clinical Research Centre, University of Surrey, Guildford, United Kingdom; Instituto Butantan, Brazil

## Abstract

The sublingual route has been proposed as a needle-free option to induce systemic and mucosal immune protection against viral infections. In a translational study of systemic and mucosal humoral immune responses to sublingual or systemically administered viral antigens, eighteen healthy female volunteers aged 19–31 years received three immunizations with a quadravalent Human Papilloma Virus vaccine at 0, 4 and 16 weeks as sublingual drops (SL, n = 12) or intramuscular injection (IM, n = 6). IM antigen delivery induced or boosted HPV-specific serum IgG and pseudovirus-neutralizing antibodies, HPV-specific cervical and vaginal IgG, and elicited circulating IgG and IgA antibody secreting cells. SL antigens induced ∼38-fold lower serum and ∼2-fold lower cervical/vaginal IgG than IM delivery, and induced or boosted serum virus neutralizing antibody in only 3/12 subjects. Neither route reproducibly induced HPV-specific mucosal IgA. Alternative delivery systems and adjuvants will be required to enhance and evaluate immune responses following sublingual immunization in humans.

**Trial Registration:**

ClinicalTrials.gov NCT00949572

## Introduction

The mucosal surface is the most common route of infection for a wide range of viral diseases and therefore inducing both mucosal and systemic immunity is a key objective of modern vaccines. The rich infiltration into the sublingual mucosa of antigen-presenting dendritic cells makes it an attractive route of immunization that avoids needles and targets the mucosal immune system [1]. Virus-Like Particles (VLP) comprising the Human Papilloma Virus (HPV) L1 major capsid protein, as well as antigens from other viruses, delivered via the sublingual route have been shown in mice to be highly immunogenic and protective against subsequent viral challenge [2], [3], [4], [5], [6]. These observations also support the idea of a “Common Mucosal Immune System” and a link between the genital tract and the systemic immune system [2], [3], [5], [6]. However, while these studies have employed antigen administration as simple sublingual liquid drops, there are characteristics of murine models which need to be considered: the murine sublingual surface is extremely rich in readily accessible dendritic cells [1]; mice are routinely anaesthetized for sublingual immunization, with possible anticholinergic effect on reducing saliva flow and antigen clearance; cholera toxin and related mucosal adjuvants have been employed to enhance responses, which may not be suitable for use in humans [7]. Sublingual immunization with non-toxic cholera toxin B subunit also induces and modulates local and disseminated responses, but this antigen is almost unique in its mucosal immunostimulating and adjuvant properties [8]. Sublingual delivery has been used for many decades in humans in desensitizing regimes involving prolonged, frequent delivery of high doses of allergens [9]. However, it is only recently that this route has been considered for delivery of prophylactic vaccine antigens, which will require far fewer doses at lower dose levels [1], [10]. We report here a preliminary human translational study to determine the character, dissemination and magnitude of systemic and mucosal immune responses to more representative antigens from a vaccine already in widespread use when administered sublingually or intramuscularly to healthy female volunteers. These results are contrasted with data from broadly similar murine studies in which HPV VLPs have been delivered sublingually as simple drops and found to be highly effective in eliciting immune response and protecting against genital HPV infection [3].

## Methods

The protocol for this trial and supporting CONSORT checklist are available as supporting information; see Checklist S1 and Protocol S1.

### Ethics Statement

Ethical Approval was obtained from the UK National Research Ethics Service, Wandsworth Research Ethics Committee reference 09/80803/77. Written informed consent was obtained from all participants after the nature and possible consequences of the study was explained. Clarification of the legal status of the study was obtained by submitting the protocol to the UK Medicines and Healthcare products Regulation Agency (MHRA) which confirmed it as a “Characterization Study” and not a Clinical Trial of an Investigational Medicinal Product (non-CTIMP/NIMP). Although not a clinical trial, we registered this study protocol on ClinicalTrials.gov (NCT00949572) prior to subject recruitment.

### Objectives

We sought to characterize, and contrast, the nature and dissemination of the immune response to sublingual or intramuscular deposition of meaningful viral vaccine antigens in humans, and to compare this with published murine studies [2], [3], [4], [5], [6]. The protocol defined no primary or secondary endpoints as this was not a clinical trial. The goal was to describe the immune response following immunization, and the study exploratory endpoint was immune response measured as several immunologic factors and assessed as change in each of these factors from pre to post immunization. The following variables were assessed before and after immunizations: (i) frequency of PBMCs secreting IgG or IgA antibodies to HPV16 L1 VLPs and whole vaccine; (ii) concentration in the serum, cervical secretions and vaginal secretions of IgG to HPV16, HPV6 and HPV18 L1 VLPs; (iii) concentration in the cervical secretions and vaginal secretions of IgA to HPV16, HPV6 and HPV18 L1 VLPs; (iv) titer in the serum, cervical secretions and vaginal secretions of neutralizing antibody to HPV16 or Bovine Papillomavirus control.

### Participants

The target recruitment defined by the protocol was 18 healthy female volunteers (in two groups: SL n = 12, IM, n = 6) aged 25–35. Subjects were all recruited at one site, St George's - University of London, London. As this was a hypothesis-generating study no formal power calculation for sample size was performed. Inclusion criteria included: provide written informed consent; in good health determined by medical history, physical examination, hematology; available for the duration of the study; if of childbearing potential, must have a negative pregnancy test before each immunization; have not donated blood in previous 3 months; eligible for free medical treatment in the UK. Exclusion criteria included: already received HPV vaccine; recent or concurrent participation in another clinical research study; recent or planned use of any investigational or non-registered product; pregnant or breast-feeding; known or suspected ongoing cervico-vaginal disease, malignancy or abnormality; positive results for Human Immunodeficiency Virus or Hepatitis B/C infection; abnormality in hematology; acute or chronic pulmonary, cardiovascular, hepatic, hematologic, renal, blood or neurological disorders, immune dysfunction, autoimmune diseases, diabetes or malignancy; recent immunosuppressive therapy; medications via vaginal route; tongue or frenulum piercings or oral jewelry; recent receipt of blood products or immunoglobulin.

### Description of Procedures or Investigations undertaken

#### Immunization

We purchased the licensed quadravalent Human Papilloma Virus (HPV) vaccine Gardasil® (Sanofi Pasteur), which contains L1-based virus-like particles (VLPs) representing four HPV types: 20 µg each of HPV types 6, 18; 40 µg each of HPV types 16 and 11 per 0.5 mL dose. VLPs are produced in yeast cells (*Saccharomyces cerevisiae* CANADE 3C-5 Strain 1895) by recombinant DNA technology and adsorbed on amorphous aluminum hydroxyphosphate sulphate adjuvant (225 micrograms aluminum per dose). All subjects received three immunizations with 0.5 mL (one standard dose) of vaccine at weeks 0, 4 and 16 (table 1) which is a recommended schedule within the flexibility of the usual 0, 1, 6 months dosing schedule for parenteral immunization. IM immunizations were given into the deltoid muscle. For SL immunization, subjects fasted (except water) for 1 hour prior to challenge, then sat in an upright position, rinsed the mouth with water and expectorated. Absorbent pads (Molnlycke ‘Dry Tips’ small) were applied over parotid duct openings bilaterally to absorb parotid saliva flow. The tongue was raised and the sublingual area gently dried by brief application of a cotton swab without inducing saliva flow from submandibular and sublingual glands. The 0.5 mL contents of a Gardasil® syringe were dispensed drop-wise to the area behind the sublingual fold bilaterally. The tongue was held in gentle opposition to the floor of the mouth for 15 minutes without swallowing, then the cotton pads removed and the subject allowed to swallow. Subjects were fasted completely for 30 minutes under observation and then requested to fast (including fluids) for a further 60 minutes after leaving the clinical site.

**Table 1:** Schedule of immunizations and sample collection.

	Week							
	0	1	4	5	8	16	17	20
Immunization	x		x			x		
PBMCs sample	x	x	x	x	x	x	x	x
Serum sample	x	x	x	x	x	x	x	x
Cervical & vaginal secretions sample	x		x		x	x		x

#### Sample collection

Table 1 shows the schedule of immunizations and sample collection. A blood sample was taken before the first immunization, at the time of the first immunization, and then on weeks 1, 4, 5, 8, 16, 17 and 20 after first immunization. Cervical and vaginal wick samples were collected at the time of the first immunization, and then on weeks 4, 16 and 20. Schedules were initiated to accommodate the subjects' menstrual cycles and a ±2 day window period was acceptable for all visits, except 1, 5 and 17 which had a ±1 day window. A protocol amendment was approved during the study to collect cervical and vaginal samples at week 8 for 3/6 subjects in IM group and 6/12 in SL group. To collect mucosal secretions a Weck-Cel surgical spear was placed either in the cervical *os* or against the vaginal wall for 2 minutes, then secretions eluted as described previously [11]. Briefly, spearheads were snipped into the top chamber of a Spin-X tube (Corning) containing 300 µL sterile filtered extraction buffer (250 mM NaCl, 16 protease inhibitor cocktail set 1 (Calbiochem) in phosphate buffered saline (PBS)) and centrifuged at 4°C for 15 minutes at 13,000 g. A repeat extraction was performed by adding additional extraction buffer to the top chamber, and then 8 µL heat inactivated fetal calf serum added to pooled secretions from each sample site, prior to separation into 200 µL aliquots and freezing at −80°C before batch analysis by ELISA as described below.

#### HPV L1 antigens used in ELISA and ELISPOT assays

HPV11 L1 VLPs were not available and no responses to HPV11 were measured. HPV 6 and 18 L1 VLPs were a kind gift of Shantha Biotechnics Ltd, India. HPV16 L1 VLPs were generated using the Bac-to-Bac® Baculovirus Expression System (Invitrogen) wherein the recombinant bacmid DNA contained an HPV16 L1 gene with a 100% amino acid sequence identity to GenBank accession numbers DQ469930 and EU118173. Recombinant HPV16-expressing baculovirus stocks were used to infect Sf21 insect cells (Invitrogen) for 72 hours at 27°C before lysis (IGEPAL® CA-630; Sigma-Aldrich) at room temperature in the presence of protease inhibitors (Complete; Roche). The cell lysate was then subjected to iodixanol gradient fractionation and gradient fractions were collected by bottom puncture and stored at −80°C. The L1 concentration and purity were visualized by SDS-PAGE stained with Colloidal Blue (Invitrogen) and analyzed using ImageJ software (U. S. National Institutes of Health, http://imagej.nih.gov/ij). VLP formation was confirmed by electron microscopic analysis of negatively staining particles (Phosphotungstic Acid; Sigma-Aldrich) adsorbed on copper grids coated with formvar (Sigma-Aldrich) and carbon.

#### Frequency of circulating L1-specific IgG and IgA spontaneously antibody secreting cells (ASCs)

The frequency of L1-specific spontaneously antibody-secreting plasmablasts was enumerated in PBMCs separated from heparinized whole blood by Ficol gradient centrifugation in an ELISPOT assay as described previously [12]. PVDF-backed 96 well plates (MAHA S45, Millipore) were coated in advance, and divided into three parts: coating buffer only in wells without any antigen as a background and nonspecific reaction control; L1 HPV16 VLPs; or Gardasil® (as other L1 antigens were not available at this time). The cell density of each sample was adjusted to 5×10^5^, 2.5×10^5^ and 1.25×10^5^/well using AIMV medium (Invitrogen, UK) containing penicillin–streptomycin. Each cell concentration was added as duplicate wells on the three antigens or uncoated parts of the plate after blocking. A 2 mg/mL PHA positive control was also added on each plate. After overnight incubation, the specific antibody-secreting cells were recognized by the goat anti-human IgG or IgA conjugated with alkaline phosphatase, and counted and analyzed by the AID EliSpot Reader System. The final results were standardized as positive cell number/1×10^5^ PBMCs plated.

#### L1-specific IgG & IgA in serum, cervical & vaginal secretion quantified by ELISA

The concentration of serum, cervical or vaginal L1-specific IgG and IgA was measured by indirect ELISA as described previously [11] using purified L1 HPV16, 6 and 18 VLPs in carbonate-bicarbonate buffer (0.05M pH 9.6) coated individually on MaxiSorp plates (Nunc). One standard curve made by a positive serum with known specific antibody concentration, and positive and negative controls were set-up on each plate, with two blank wells on each plate to monitor background. Serum, cervical and vaginal samples were diluted 1/200 or 1/4 with 0.05% PBS–T20, respectively, and any sample with OD value above the upper limit of the standard curve was further diluted and re-assayed. The specific IgG or IgA was recognized by goat anti-human IgG or IgA conjugated with peroxidase. The concentrations of specific antibodies were measured by an Emax MAXLine Microplate reader at 650 nm after the TMB liquid substrate developed. The raw OD data was analyzed using SoftMax® software.

#### Functional antibody in serum and cervical and vaginal secretions measured by in vitro pseudovirus neutralization assay

The HPV16 pseudovirus neutralization assay [13] was carried out as previously described [14] and included Bovine Papillomavirus (BPV) as a control for non-specific antibody effects. As a control, the WHO International Standard for HPV16 antibodies, IS16 (code: 05/134; 10 IU/mL; National Institute for Biological Standards and Control, UK; [15]) demonstrated type-specific neutralization of HPV16 at levels consistent with natural infection (median titer 138 [inter-quartile range 115–148]; n = 3).

### Statistical methods

As this was not a clinical trial, no randomization was performed and no safety data (adverse events) were solicited, no primary or secondary endpoints were specified. Subjects were allocated to one of two sequential cohorts: “IM” who received all immunizations via the intramuscular route (n = 6); and “SL” who received all immunizations as sublingual drops (n = 12). Subjects were not randomized as we wished to develop and evaluate B cell assays carried out on fresh blood samples by recruiting the first subjects into the intramuscular delivery group (and for whom measurable antibody secreting cells (ASCs) were likely to be seen). As the immunization routes could not be blinded the study was not blinded. There were no protocol deviations. As this hypothesis-generating study was not powered to detect significant differences between groups or between time points, no statistical testing was performed and descriptive statistics only are presented.

## Results

### Subjects enrolled

**Figure 1:**
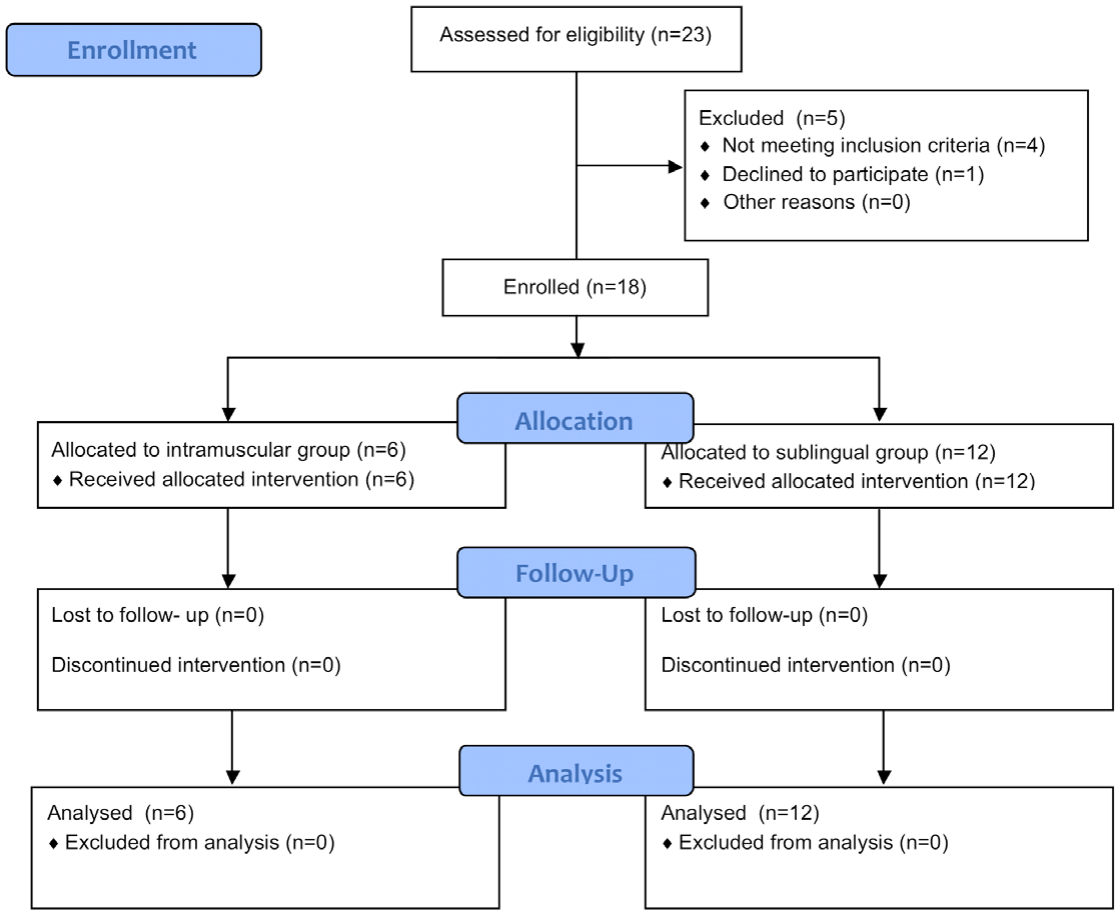
CONSORT diagram.

Eighteen female subjects aged 19–31 years (IM group mean 24.2, median 25; SL group mean 26.3, median 27.5) were enrolled and completed the protocol. There were no protocol deviations (figure 1).

### Circulating Anti-L1 HPV16 B cell responses measured by ELISPOT

**Figure 2:**
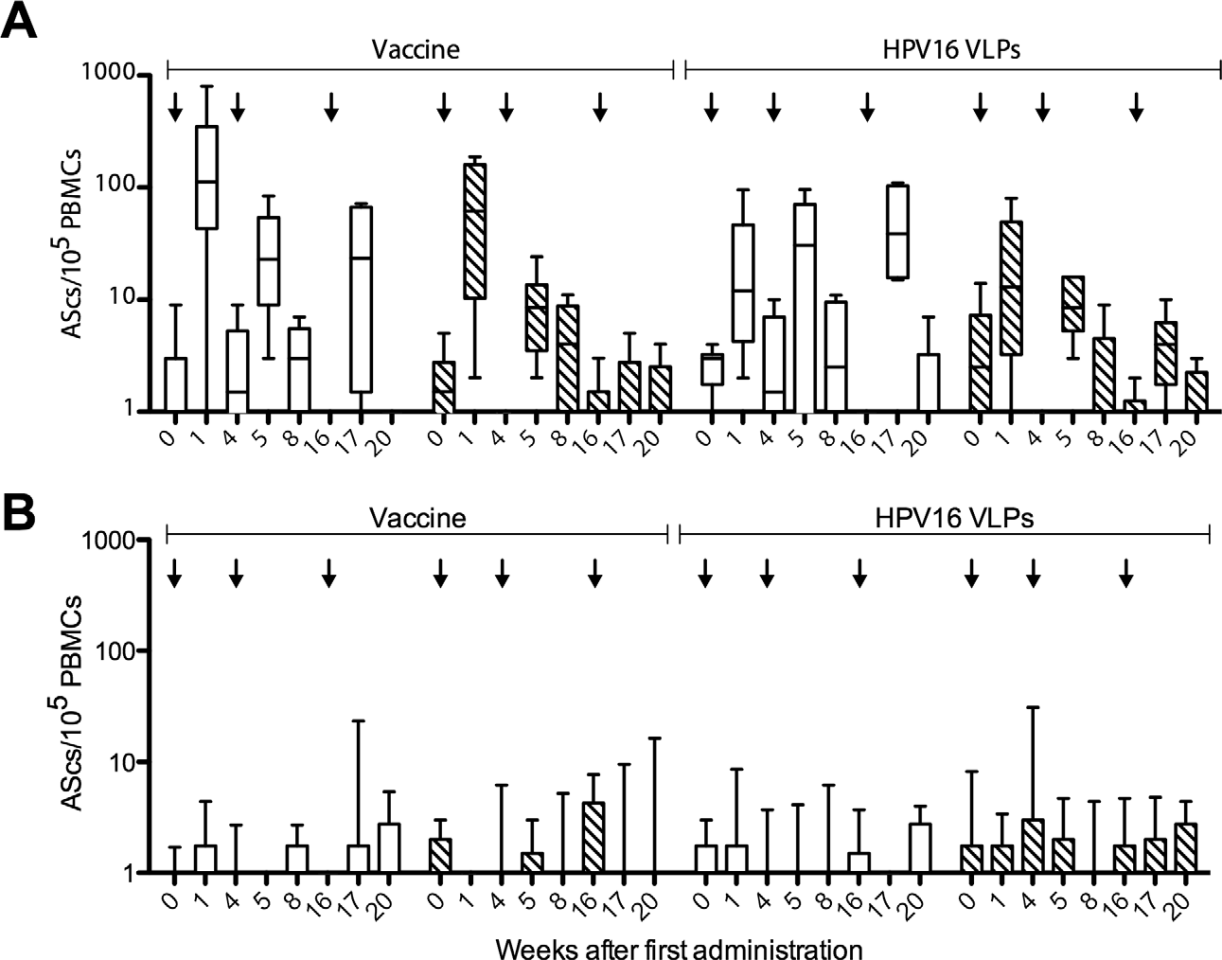
Circulating antibody secreting cell responses. The Y axis values indicate the group median frequency of antibody secreting cells (ASCs) per 10^5^ PBMCs plated, secreting IgG (white bars) or IgA (hatched bars) against Gardasil vaccine or L1 HPV16. Panel A: subjects immunized intramuscularly. Panel B: subjects immunized sublingually. Arrows indicate immunizations. Box: 25th to 75th percentiles, whiskers: 10 to 90 percentiles.

HPV16 L1 VLPs and whole vaccine (VLP HPV 6, 11, 16 and 18) were used as a coating antigens. From previous studies using oral, nasal or intramuscular immunization [12], [16], a transient increase in the frequency of cells spontaneously secreting ant-L1 IgG and IgA was expected, peaking around 7 days after each immunization (on weeks 0, 4 and 16) and then falling back to baseline, reflecting the generation and maintenance kinetics of plasmablasts. An increase in IgG and IgA antibody secreting cell (ASC) frequencies was seen after IM immunizations (figure 2), but not after SL immunization.

### Anti-L1 HPV6, HPV16 and HPV18 serum & cervico-vaginal IgG & IgA responses measured by ELISA

**Figure 3:**
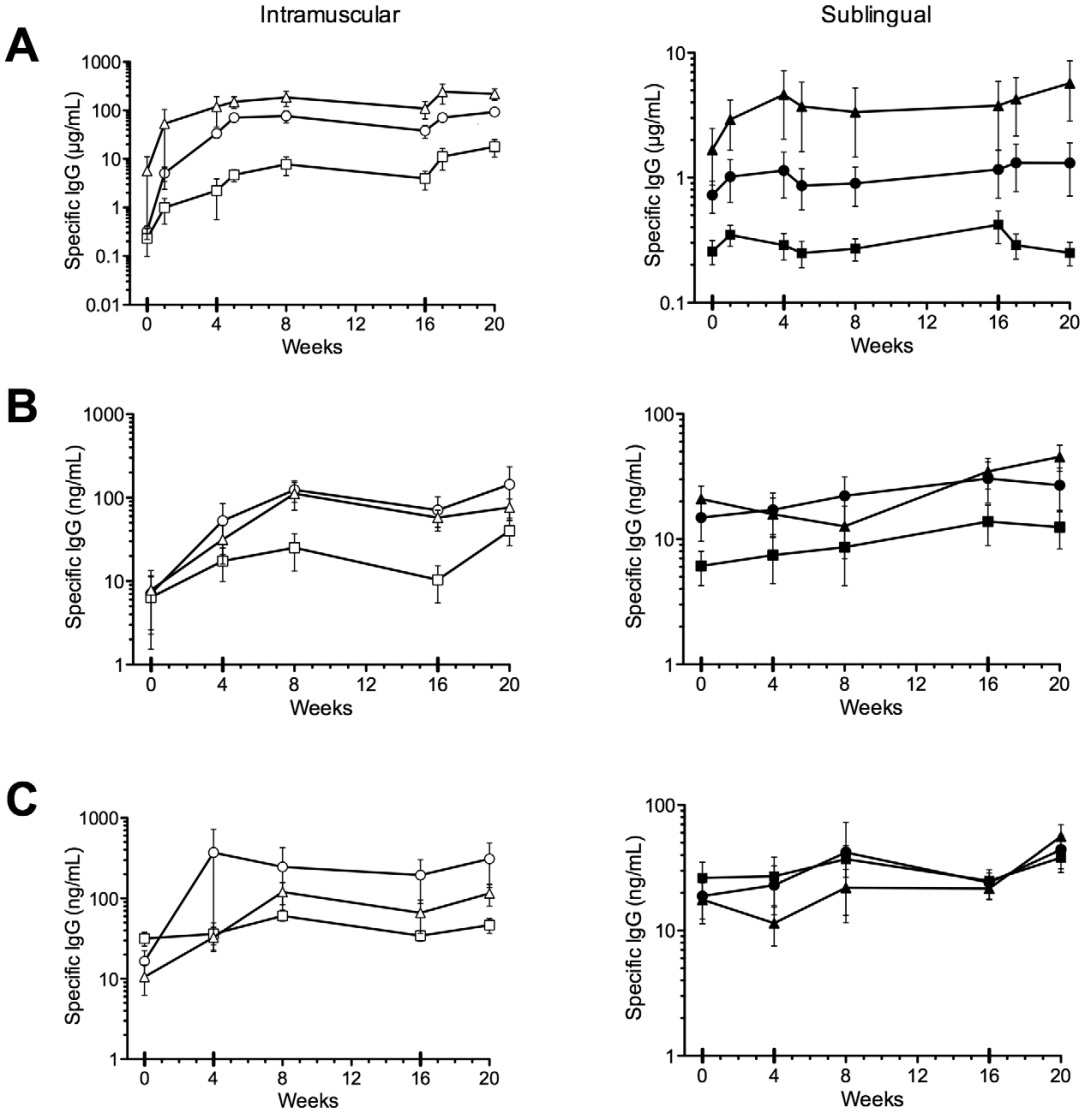
Serum, cervical and vaginal IgG responses. The Y axis values (note different scales) indicate group mean anti-L1 HPV6 (circles), HPV16 (triangles) and HPV18 (squares) IgG concentration in serum (panel A), cervical secretions (panel B) or vaginal secretions (panel C), for subjects immunized intramuscularly (left, open symbols), or sublingually (right, closed symbols). Error bars SEM.

**Figure 4:**
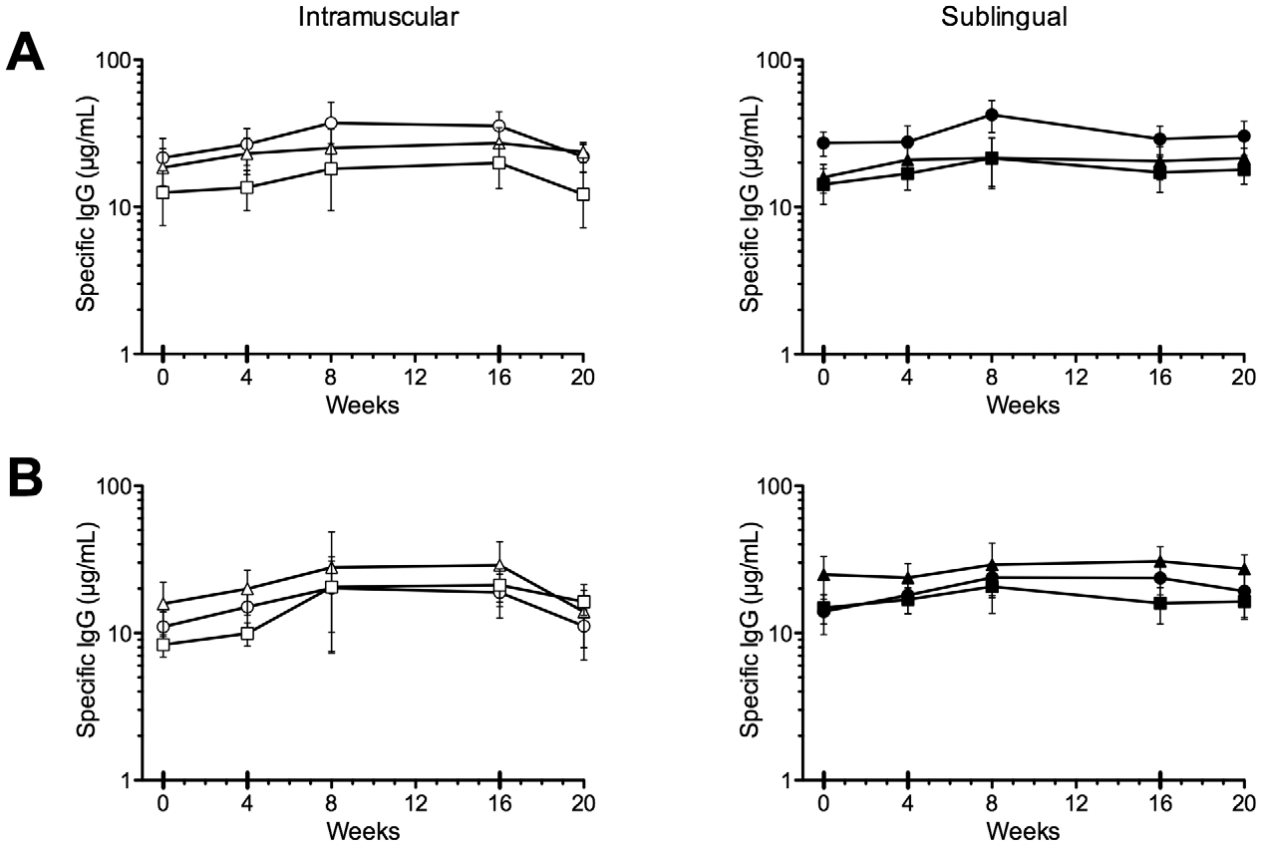
Cervical and vaginal IgA responses. The Y axis values indicate group mean anti-L1 HPV6 (circles), HPV16 (triangles) and HPV18 (squares) IgG concentration in cervical secretions (panel A), or vaginal secretions (panel B), for subjects immunized intramuscularly (left, open symbols), or sublingually (right, closed symbols). Error bars SEM.

The kinetics of the anti-HPV L1 VLP antibody response in serum and in cervico-vaginal secretions was measured before and at various time points after each immunization using an antigen-specific antibody binding ELISA, with purified L1 HPV16, 6 and 18 VLPs as coating antigens. As expected, IM immunization induced an increase in serum anti-HPV6, HPV18 and HPV16 L1 VLP IgG from baseline (figure 3). IM immunization also induced an increase in cervical anti-HPV6 and HPV16 L1 VLP IgG, and to a lesser extent in anti-HPV18 L1 VLP IgG. An increase in vaginal anti-HPV6 and HPV16 L1 VLP IgG was seen, but not anti-HPV18 L1 VLP IgG. SL immunization induced an increase in anti-HPV16 L1 VLP IgG in serum, cervical secretions and vaginal secretions, and a slight increase in cervical anti-HPV18 L1 VLP IgG. However, the serum anti-HPV16 L1 VLP IgG at week 20 after IM immunization was 219 µg/mL (SEM ±57.3, a 38.9-fold rise from week 0), compared with 5.73 µg/mL (±2.9, 3.4-fold rise) after SL immunization. In contrast, relative levels of specific IgG in mucosal secretions were not as dissimilar as in serum: mean 45.5 ng/mL (±10.8, 2.2-fold rise) in cervical secretions after SL immunization, 76.7 ng/mL (±19.6, 9.8-fold rise) after IM immunization. Similarly, in vaginal secretions the values were 56.1 ng/mL (±13.8, 3.2-fold rise) and 115.5 ng/mL (±34.9, 10.9-fold rise) for SL and IM immunizations, respectively. IgA responses in secretions were variable at all time points (figure 4).

### Neutralization of HPV16 by serum and cervical and vaginal secretions

The detection of antibodies capable of neutralizing HPV16 pseudoviruses was examined using serum and cervical and vaginal samples taken at week 0 and 20 (4 weeks post last immunization) for all subjects (table 1). Some intermediate time points were also evaluated for selected subjects (data not shown). The control Bovine Papillomavirus (BPV) was used as a control for non-specific antibody reactivity. All subjects had undetectable virus neutralization titers in mucosal secretions at week 0 (table 2), but 2/6 and 1/12 subjects in the IM and SL groups, respectively, had detectable serum virus neutralizing activity at week 0, which is in line with the ∼12% prevalence estimates for previous HPV16 infection expected in this population [17], [18]. IM immunization induced or boosted serum neutralizing antibodies in all subjects at week 20, and very low level neutralizing titers also appeared in mucosal secretions at week 20 after IM immunization in the 3/6 subjects with the highest serum neutralizing titers. Sublingual immunization did not induce any neutralizing activity in mucosal secretions. However, sublingual immunization did boost pre-existing serum neutralizing activity in one subject (013) to a level similar to that seen in subjects without pre-existing neutralizing activity who received IM immunization and induced weak serum neutralizing titers in two others (015, 023). This suggests that, unlike IM immunization, while SL immunization may not be very effective at priming the immune response it may be able to boost pre-existing immunity.

**Table 2:** HPV16 pseudovirus neutralization by serum and genital antibodies.

		HPV16 Neutralization Titer using in indicated sample					
		Cervix		Vagina		Serum	
Immunization Route	Subject ID	Week 0	Week 20	Week 0	Week 20	Week 0	Week 20
Intramuscular	001	-a	-	-	-	-	4,367
	004	-	-	-	26	-	34,255
	005	-	-	-	-	-	4,055
	017	-	-	-	-	-	3,856
	018	-	51	-	39	189	61,743
	020	-	42	-	53	5,086	44,494
Sublingual	006	-	-	-	-	-	-
	007	-	-	-	-	-	-
	008	-	-	-	-	-	-
	009	-	-	-	-	-	-
	010	-	-	-	-	-	-
	011	-	-	-	-	-	-
	013	-	-	-	-	213	2,249
	014	-	-	-	-	-	-
	015	-	-	-	-	-	632
	019	-	-	-	-	-	-
	021	-	-	-	-	-	-
	023	-	-	-	-	-	92

a ‘-’, indicates reciprocal neutralization titers <20. All samples tested negative for neutralizing antibodies against the control Bovine Papillomavirus, BPV.

## Discussion

Parenteral immunization with vaccines containing HPV L1 VLPs and formulated using alum and/or TLR agonist adjuvants is highly effective at inducing both serum and mucosal antibodies, and conferring long-lasting protection against HPV infection by the homologous or related HPV genotypes [19], [20], [21], [22]. Several murine models have shown that simple drops placed under the tongue can induce functional antibody and T cell responses to viruses such as Herpes simplex virus (HSV), influenza, Human Immunodeficiency Virus (HIV) and HPV, and protection against genital challenge with HSV and HPV [2], [3], [4], [5], [6]. The possibility to develop a needle-free sublingual human vaccine, specifically targeting the induction of mucosal immunity and applicable to a wide range of genital viral infections is compelling [1]. However, these murine models often incorporate features that are not compatible with real-world human vaccine strategies, such as the use of anti-cholinergic anesthetics that may block saliva flow, and mucosal adjuvants based on cholera toxin-related proteins that are unsafe in humans when given nasally [7]. Sublingual desensitization regimes use frequent, prolonged high doses of allergens [9]. Non-toxic cholera toxin B subunit antigens induce disseminated antibody responses after sublingual immunization, but this molecule has intrinsic mucosal immunogenicity and adjuvanticity not seen in the majority of protein antigens [8], and like allergens may therefore not be representative of real word sublingual human vaccine antigens. We carried out a preliminary translational study to characterize immune responses to the sublingual or parenteral administration of a viral antigen that is already in widespread use as a human vaccine [23], and therefore representative of the human application of sublingual immunization. As this was the first use of this vaccine sublingually in humans we followed a 0, 1, 4 month prime-boost schedule which is an acceptable schedule for IM immunization using Gardasil®.

We observed that sublingual immunization generally induced a similar pattern of immune responses to intramuscular, but at much lower magnitudes. An increase in serum anti-HPV16 L1 VLP IgG was detected after both IM and SL immunization (figure 3). However, the serum anti-HPV16 L1 VLP IgG at week 20 after IM immunization was ∼38 times higher than after SL immunization. Similarly, while IM immunization was able to both prime or boost serum virus neutralizing activity in all subjects, sublingual immunization could only boost serum neutralizing activity in a subject with pre-existing activity at week 0, and induce low levels of serum neutralizing antibody in two other subjects with undetectable neutralizing activity at day 0. This suggests that with optimization, the sublingual route may have a role in boosting pre-existing immunity induced by another route, and is capable of inducing functional antibody.

One of the potential translational advantages proposed for mucosal immunization is that it appears to specifically induce mucosal immunity [10]. However, while IM immunization was capable of inducing measurable virus neutralizing activity in cervical and/or vaginal secretions in 3/6 subjects (concomitant with high serum neutralizing titers suggesting transudation of serum IgG), no mucosal virus neutralizing activity was induced by SL immunization in any subject. Similarly, while IM immunization induced increases in mucosal anti-HPV6 and HPV16 L1 VLP IgG, sublingual immunization only induced an increase in mucosal anti-HPV16 L1 VLP IgG. However, it is intriguing that relative levels of specific IgG in mucosal secretions were not as dissimilar as in serum with only ∼1.7-fold higher levels in cervical secretions after IM immunization compared with SL immunization. Similarly, in vaginal secretions IM immunization gave ∼2-fold higher levels. This can be interpreted as SL immunization preferentially favors mucosal over systemic responses, or that neither route is very efficient at inducing mucosal immunity. Mucosal IgA responses were infrequent, low level, transient and extremely variable after either SL or IM immunization, despite HPV-specific IgA ASC responses after IM immunization. The kinetics of the ASC response to sublingual immunization is not well defined in humans and it is possible that we missed the response to SL immunization. Additionally, although ASC responses to the first IM immunization appeared higher than subsequent immunizations, previous studies [16] have shown a shift to a slightly earlier timing of the peak response to booster immunizations (around day 5), which may explain the apparent fall in frequency measured 7 days after the booster immunizations.

Due to the considerable volume of saliva produced despite covering the parotid ducts, it is highly likely that some sublingually administered VLPs would have been removed from the mucosal surface within minutes of application. To be effective, extensive optimization of sublingual delivery will be required to improve penetration of the sublingual mucosa of humans, perhaps by making use of mucoadhesives or other delivery systems designed to resist salivary degradation [24]. In addition, the size of VLPs and VLP-alum aggregates may have restricted access across the mucosal barrier. Manipulation of the particle size may therefore optimize responses. However, despite these caveats, some HPV-specific immunity was generated *de novo* in 2/12 subjects whose day 0 serum neutralizing antibody titer was below the cut-off in our assay, and boosted the day 0 titer in another subject. These observations are encouraging for potential future vaccine strategies based upon sublingual delivery. It is also possible that the bivalent vaccine, Cervarix®, which makes use of a TLR-4 agonist in the vaccine formulation and has been shown to induce higher levels of HPV-specific antibodies when administered parenterally, may have induced significantly higher antibody levels [25], especially if combined with mucosal delivery systems that enhance sublingual contact times [24]. We allowed subjects to swallow retained saliva, and theoretically this may have allowed some immune responses to be induced in the small intestine. However, it is unlikely that 20–40 µg of VLPs would survive gastric acid, and indeed even the uniquely immunogenic mucosal antigen and adjuvant cholera toxin or its B subunit given at doses of 1–5 mg requires buffering with bicarbonate solution to retain immunogenicity via the oral route in humans [26], [27].

In conclusion, this preliminary translational human study indicates that, in marked contrast to murine studies, SL delivery of a representative virus vaccine antigen formulated with alum is only modestly immunogenic in humans. This route can, however, induce low level serum and mucosal antibodies, and functional serum neutralizing antibody. The observation that SL immunization could boost pre-existing serum neutralizing activity also points to the possible use of IM prime/SL boost schedules. For this approach to be advanced, the next steps require significant optimization of the SL delivery system for human use, and the investigation of optimal SL-IM prime-boost schedules. Once this is achieved the benefits of sublingual delivery on the character, magnitude and dissemination of responses should be compared in clinical trials.

## Supporting Information

Protocol S1 Trial Protocol.
(PDF)

Checklist S1 CONSORT Checklist.
(DOC)
