# Supplementary material for: Systemic and Mucosal Immune Responses to Sublingual or Intramuscular Human Papilloma Virus Antigens in Healthy Female Volunteers
Source: PLoS One. 2012 Mar 16;7(3):e33736. doi: 10.1371/journal.pone.0033736 (PMC3306286; doi:10.1371/journal.pone.0033736)
Supplement: Protocol S1 — Trial Protocol. (PDF) [file pone.0033736.s001.pdf]

St George's
University of London

CLINICAL STUDY PROTOCOL

Characterisation of human disseminated cellular and humoral immune responses following sublingual or intramuscular deposition of antigens

Short title: Measuring responses to sublingual antigens

Study Identification: SG09-EN01

REC number: 09/80803/77

Version number: 1.04.2c

Date: 21 APRIL 2009 / 06 August 2009
Amendment 2.   27 Jan 2010
Amendment 3   23rd Feb 2010

This study will be conducted in accordance with ICH GCP Guidelines (Directive CPMP/ICH/135/95) and the Declaration of Helsinki (1964) and subsequent amendments.

SYNOPSIS

**Title**	Characterisation of human disseminated cellular and humoral immune responses following sublingual or intramuscular presentation of antigens
**Short Title**	Immune response to sublingual antigens
**Sponsor**	St. George's University of London
**Principal Investigator**	Prof. David J. M. Lewis
**Planned Study Dates**	October 2009 - September 2010
**Study type**	Physiology study with challenge agents
**Objectives**	To explore in humans the:<br>1. Concentration and isotype profile of antigen-specific antibody in serum and cervico-vaginal secretions<br>2. Frequency and isotype profile of antigen-specific antibody secreting cells in blood<br>3. Frequency and expression profile of mucosa-associated homing, memory and regulatory markers on antigen-specific T cells in blood in response to *in vitro* antigen stimulation<br>4. Profile of cytokine secretion by peripheral blood mononuclear cells in response to *in vitro* antigen stimulation measured by ELISA<br>following presentation of an immunological challenge agent via sublingual or axillary lymph nodes
**Challenge Agents**	Purified, recombinant L1 protein antigens from Human Papilloma Virus serotypes 6, 11, 16 & 18 in the following amounts:<br>Human Papillomavirus Type 6 L1 protein 20 micrograms<br>Human Papillomavirus Type 11 L1 protein 40 micrograms<br>Human Papillomavirus Type 16 L1 protein 40 micrograms<br>Human Papillomavirus Type 18 L1 protein 20 micrograms<br>adsorbed on amorphous aluminium hydroxyphosphate sulphate adjuvant (225 micrograms Al)
**Study design**	• Physiology study using challenge agents, non-CTIMP<br>• Open label, non-randomised, hypothesis generating study
**Sample size**	EIGHTEEN subjects in all, in two groups:<br>(1) SIX receiving challenge agents by intramuscular injection of deltoid muscle<br>(2) TWELVE receiving challenge agents sublingually
**Study population**	Healthy female adult volunteers aged 18-35

**Route of Administration**	1. Intramuscular (deltoid injection): 6 subjects<br>2. Sublingual (as drops under the tongue): 12 subjects<br>Given on three occasions at 0, 1 and 4 months
**Duration of Treatment**	The entire duration of the study will be 20 weeks (up to 24 weeks including screening period).<br>During this period subjects will make a total of 9 outpatient visits to the clinical site
**Primary & Secondary endpoints**	No primary and secondary endpoints are defined in this hypothesis-generating study.<br>A number of exploratory variables will be measured.
**Exploratory variables**	1. Concentration, neutralising activity and isotype profile of antigen-specific antibody in serum and cervico-vaginal secretions measured by ELISA and/or LUMINEX assay and virus neutralisation assay.<br>2. Frequency and isotype profile of antigen-specific antibody secreting cells in blood measured by ELISPOT assay<br>3. Frequency and expression profile of mucosa-associated homing, memory and regulatory markers on antigen-specific T cells in blood in response to *in vitro* antigen stimulation measured by Flow Cytometry (FACS) and CFSE proliferation assay<br>4. Profile of cytokine secretion by peripheral blood mononuclear cells in response to *in vitro* antigen stimulation measured by ELISA
**Safety evaluations**	None, not a CTIMP
**Efficacy evaluation**	None, not a CTIMP

Signatures

PROTOCOL APPROVED BY

Prof. David JM Lewis, St George's University of London
Signature ______________   Date 22 FEB 2010

I AGREE TO CONDUCT THE STUDY IN ACCORDANCE WITH THE INFORMATION CONTAINED IN THIS STUDY PROTOCOL:

Principal Investigator, St George's University of London
Prof. David JM Lewis
Signature ______________   Date 22 FEB 2010

Table of Contents

1 General Information 6
2 Study Background 8
2.1 Research Background 8
2.2 Challenge Agents 10
2.3 Objectives and Purpose 10
2.4 Preclinical and clinical data 12
2.5 Population 12
2.6 Dose Rationale 13
2.7 Risk/Benefits 14
2.7.1 Potential Risks 14
2.7.1.1 General risks 14
2.7.1.2 Intramuscular injection 14
2.7.1.3 Topical sublingual application 15
2.7.2 Potential Benefits 15
2.7.2.1 Intramuscular injection 15
2.7.2.2 Topical sublingual administration 16
2.8 Subject population(s) for analysis 16
2.9 Study design/type 17
2.9.1 Schedule of visits and procedures 17
2.9.2 Acceptable Visit Windows 17
2.9.3 Details of study visits 18
Visit 1: Eligibility check 18
Visits 2, 4 and 7: Antigen delivery and immunology response follow-up 18
Visits 3, 5, 6 and 8: Immunology response follow-up 18
Visit 9: Final immunology response follow-up 18
2.10 Data Identification 19
2.11 Primary and/or Secondary Study Endpoints 19
2.12 Randomisation 19
2.13 Maintenance of randomisation codes 19
2.14 Inclusion criteria 20
2.15 Exclusion criteria 20
2.16 Subject withdrawal criteria 21
2.17 Study treatment 22
Name of challenge agents 22
Qualitative and quantitative composition 22
Dosage regimen 22
Formulation 22
Excipients 22
Incompatibilities 22
Shelf life 22
Special precautions for storage 22
Nature and contents of container 22
Regulatory requirements 22
2.18 Duration 23
2.19 Treatment of subjects 23
2.20 Discontinuation 23
2.21 Challenge Agents Accountability 24
2.22 Accountability procedure 24
2.23 Monitoring subject compliance 24
2.24 Permitted medication 24
2.25 Efficacy parameters 24
2.26 Method and timing for efficacy parameters 24
2.27 Safety parameters 24
2.28 Adverse event reporting 24
2.29 Adverse event follow-up 25
2.30 Statistical methods 25
2.31 Subject analysis 25
2.32 Method and timing for safety parameters 25
2.33 Termination criteria 25
2.34 Ethical considerations 26
2.35 QC & QA 26
2.36 Deviation reporting 27
2.37 Subject analysis 27
2.38 Data storage 27

2.39 Finance and insurance arrangements 27
2.40 Publication rights 27
2 Supplementary Information 28
2.41 Literature 28
2.42 Supplementary details 29
Appendix 1: Standard Operating Procedure for sublingual application of Gardasil 30
Appendix 2: Characteristics of staff delegated to administer challenge agents 31

1 General Information

**Protocol title**	Characterisation of human disseminated cellular and humoral immune responses following sublingual or intramuscular presentation of antigens
**Protocol title acronym**	SG09_EN01
**Sponsor**	St George's University of London
**Host site**	St George's University of London
**Name(s), address(es) and telephone number(s) of clinical laboratory (ies) & other medical and/or technical department(s) and /or institutions involved in the study**	Abraham Roodt,<br>Clinical Trials and Data Manager<br>The Doctors Laboratory<br>60 Whitfield Street<br>London<br>W1T 4EU<br>Tel: +44 (0)20 7307 7373<br><br>Zhiming Huo MD PhD<br>St. George's Vaccine Institute<br>Centre for Infection<br>St. George's - University of London<br>Cranmer Terrace<br>London SW17 0RE<br>Tel: 0 20 87 25 32 11<br><br>Simon Beddows Ph.D.<br>Principal Scientist<br>HPV R&D Section Head<br>Virus Reference Department<br>Centre for Infections<br>Health Protection Agency<br>61 Colindale Avenue<br>London NW9 5HT<br>Tel: +44 (0) 20 8327 6169<br>Fax: +44 (0) 20 8200 1569<br><br>R. Karl Malcolm BSc (Hons) PhD CChem MRSC<br>PGCHET Senior Lecturer in Pharmaceutics<br>School of Pharmacy<br>Medical Biology Centre<br>97 Lisburn Road Belfast<br>BT9 7BL Northern Ireland<br>T: +44 (0)28 9097 2319<br>E: k.malcolm@qub.ac.uk

**Name, title, address and telephone number(s) of Principal Investigator for the study**	Prof. David JM Lewis MD<br>St. George's Vaccine Institute<br>Centre for Infection<br>St. George's - University of London<br>Cranmer Terrace<br>London SW17 0RE<br>Email: sgf300@sgul.ac.uk<br>Tel: 0 20 87 25 58 26/7/8/9<br>Fax: 0 20 87 25 34 87

2 Study Background

2.1 Research Background

Please provide any relevant background information to support the research area/disease

This study builds on and extends a series of studies we have undertaken using licensed vaccines as safe human challenge agents to induce an immune response that can then be characterised. Using this approach we have characterised the profile of B and T cell responses following oral and nasal administration and intramuscular injection of model antigens[1-4].

It is well recognised that uptake and presentation of antigens either locally or in lymph nodes draining mucosal surfaces such as gut, respiratory, or genital tracts, often induces an immune response with a different profile that when the same antigens are presented in systemic lymph nodes following intramuscular injection[5]. In our previous studies we found for example that while oral delivery of antigen induces mainly an IgA response amongst circulating B cells, and surface markers indicating a gut homing tendency, nasal administration of antigens induces a mixed IgG and IgA response, and both systemic and mucosal homing markers[1-4]. This, and others' work, has contributed the concept of a "common mucosal immune system" (CMIS) distinct from the systemic immune system. Injected (intramuscular or subcutaneous) antigens tend to interact with the systemic immune system, induce IgG, and lymphocytes with surface markers (such as L-selectin) that home to systemic but not mucosal tissue. In contrast, application of antigen to a mucosal surface spreads responses throughout the mucosal immune system by the expression of mucosal-homing markers such as (α4β7 integrin for gut and α4β1 integrin and CCR10 to genital tract[6]). There are limitations to dissemination within the CMIS: for example nasal immunisation preferentially induces immunity in the respiratory and genital tracts, whereas rectal immunisation preferentially induces immunity in the large bowel[5]. A better understanding of how the common mucosal immune system is "wired-up", and an improved knowledge of the cell surface markers that guide lymphocytes to mucosal or systemic sites, will be of value in better understanding inflammatory and immune-mediated diseases such as post reactive arthritis, and designing novel vaccines and vaccine delivery systems.

Whereas 'oral' delivery of antigens has tended to involve the ingestion, swallowing and presentation of antigen to small bowel immune induction sites, interest has focussed recently on sublingual delivery of antigen. The sublingual mucosa is rich in dendritic cells that can take up antigens and present them to the mucosal immune system[7]. It has been known for many years that repeated sublingual administration (usually daily application for many weeks) of high doses of *allergens* (such as grass pollen) is safe, and induces a non-allergic immune response, which is as clinically effective in reducing allergic responses to the same allergens as desensitisation by repeated subcutaneous injections[7]. Different patterns of cell surface markers associated with regulatory function, and cytokine secretion in response to stimulation have also been observed after nasal and sublingual routes of allergen delivery[8], which is an area we wish to explore using the pathogen-derived antigen challenge agents in this study.

While the conversion of an allergic-type of immunity to a non-allergic type of immunity appears to require frequent sublingual application of high doses of allergens, it has recently been shown that only a few sublingual exposures of pathogen-derived *antigens* can induce protective immunity. For example, sublingual administration of inactivated influenza virus to mice on two occasions induced both systemic and mucosal antibody responses and conferred protection against a lethal intranasal challenge with influenza virus[9]. These responses could be enhanced when cholera and E. coli toxin-derived adjuvants were co-administered[9]. Cholera toxin subunit B itself induces systemic and genital tract immune responses in mice[10] and humans (J Holmgren personal communication) after sublingual

immunisation. However, molecules such as cholera toxin are somewhat unusual in that they are potent immunogens and mucosal adjuvants in their own right, with powerful effects on immune cells that polarise and alter immune response profiles[5], and so are not very representative of protein antigens in general.

It is therefore of interest to observe the profile of more representative protein *antigens* following sublingual delivery, and to compare this with our and others' experience with nasal, oral and injected delivery of antigens. We have selected the L1 protein of Human Papilloma Virus (HPV) as a representative protein antigen to use as a challenge agent to induce immune responses disseminated within the CMIS, and the systemic immune system, which can then be studied and characterised. Each strain of HPV has a unique L1 protein antigen: strains 16 and 18 are associated with cervical cancer in women, and strains 6 and 11 with anogenital warts in both sexes.

The HPV L1 antigens have been selected from a number of potential immunological challenge agents, as they possess several advantageous characteristics for a human immunological challenge agent:

1. They are antigens from the capsid of a human pathogenic virus that infects the genital tract, and therefore representative of a wide number of viral protein antigens.
2. They are available as a pure preparation, suitable for human use, in the licensed HPV vaccine "Gardasil".
3. Their safety via injection has been demonstrated in numerous clinical trials of HPV vaccines, and the UK Dept of Health is engaged in a mass immunisation campaign employing HPV L1 antigen vaccines in healthy young adult women.
4. The optimum safe and immunogenic human dose of L1 antigens has been determined after injection, which provides a guide to the dose suitable for sublingual application.
5. They spontaneously form "Virus Like Particles" in the "Gardasil" preparation. These larger structures are expected to be more readily taken up by sublingual dendritic cells than small proteins or peptides, and so increase the probability of a detectable immune response to the challenge.
6. They are adsorbed onto alum, which has been used for decades in many licensed vaccines. This will aggregate and is expected to further enhance dendritic cell uptake.
7. They are known to reliably induce both serum and genital tract antibody responses in women after injection, and assays to measure responses in genital tract secretions are described [11]. This ensures we will have a reliable positive response group, which we will recruit first to enable assay development for subsequent application to the sublingual challenge group in which immune responses are expected to be lower and less frequent.
8. The "Gardasil" preparation has the advantage of containing 4 different L1 antigens (from strains 6, 11, 16 and 18). Seroepidemiology of women aged 23 (the median age of subjects generally recruited into our studies) in England[12] shows that around 45% already have immunity to at least one of the 4 antigens, but only 15% have immunity to two or more. This means that in our sample we will expect half the subjects not to be immune to any of the antigens, in whom we can study priming and boosting responses to a neoantigen, which is the most interesting aspect of this study - as previous genital tract infection may bias the subsequent response to the sublingual immune challenge.

   However, we will also have around half of the subjects in whom we can simultaneously study responses to recall antigens that they have seen before (thereby increasing our prospects of seeing any response at all to sublingual challenge) and neoantigens. While this hypothesis generating study is not powered to analyse subgroup responses to the 4 antigens in detail, by determining subject's serostatus at entry it will be interesting to see if there is any suggestion that prior genital tract infection modulates the response to

cross-reacting antigens. Prior genital infection is essentially a mucosal immunological prime, which may be boosted by the mucosal (sublingual) or parenteral (intramuscular) boost in different ways. It has recently been shown that around 1% of young women have oral infection with HPV[13], which might theoretically modulate the immune response. However the low prevalence is unlikely to significantly affect the number of subjects being studied. Any observed differences in immune responses between subjects seropositive or seronegative at entry could be further investigated in larger studies.

2.2 Challenge Agents

Please provide details of challenge agents(s) to be used in the study (including name, manufacturer, country of manufacture, form)

The challenge agents will consist of purified, recombinant L1 protein antigens from Human Papilloma Virus serotypes 6, 11, 16 & 18.

A single, 0.5 mL pre-filled syringe of "Gardasil" Human Papillomavirus Vaccine [Types 6, 11, 16, 18] (Recombinant, adsorbed) will be used for each challenge, containing:

Human Papillomavirus Type 6 L1 protein 20 micrograms

Human Papillomavirus Type 11 L1 protein 40 micrograms

Human Papillomavirus Type 16 L1 protein 40 micrograms

Human Papillomavirus Type 18 L1 protein 20 micrograms

L1 proteins are in the form of virus-like particles produced in yeast cells (Saccharomyces cerevisiae CANADE 3C-5 (Strain 1895)) by recombinant DNA technology, adsorbed on amorphous aluminium hydroxyphosphate sulphate adjuvant (225 micrograms Al), together with the following excipients: Sodium chloride, L-histidine, polysorbate 80, Sodium borate, Water for injections.

Each dose is presented as a suspension in a pre-filled syringe.

The Marketing Authorisation Holder is Sanofi Pasteur MSD SNC, 8 rue Jonas Salk, F-69007 Lyon, France. Vaccines will be purchased through an approved supplier in the UK.

Syringes will be stored in the Pharmacy Area of the Vaccine Institute in a refrigerator (2°C - 8°C).

Vaccine Institute SOPs for receipt, storage, accountability and reconciliation will be followed.

2.3 Objectives and Purpose

Please Insert a detailed description of the objectives and the purpose of the study

The purpose of this hypothesis-generating study is to explore in healthy humans the profile of the mucosa-associated and systemic immune systems' response to pathogen-derived protein antigens.

We will induce an immune response in healthy volunteers by the application of immunological challenge agents to the mucosal immune system (locally or via the sublingual lymph nodes), and the systemic immune system (probably via axillary lymph nodes). This immune response will then be characterised by a number of immune assays, and any differences and similarities in the pattern of responses induced by the two systems explored.

We will apply techniques that we have developed and published in previous studies of oral,

nasal and intramuscular challenge, as well as developing new techniques to characterise T cell responses and regulatory, memory and genital tract-associated surface markers.

Initially we will recruit and challenge subjects via the intramuscular route, as this is known to reliably induce systemic and vaginal secretion antibody responses. This will enable us to develop exploratory cellular assays. Once we are confident that the novel assays are performing with acceptable accuracy and reliability, we will recruit subjects to receive sublingual challenge, as this route of challenge is expected to give lower level and less frequent responses due to reduced antigen access across the sublingual mucosa.

Blood will be taken at several time-points for serum antigen-specific antibody levels and enumeration and characterisation of antigen-specific T and B cells using well-established techniques. We will collect cervical and vaginal secretions using well-established techniques within the Vaccine Institute in which secretions are collected onto clinical ophthalmic sponges by gentle application against the wall of the vagina and cervix under direct speculum examination. Secretions are extracted by centrifugation into a preservative buffer that prevents protease degradation, prior to analysis for antigen-specific antibody in ELISA or Luminex assay and neutralisation assay.

As this is a hypothesis generating study it is not expected that we will be able to characterise all the parameters, in all the assays, in all the subjects. The core assays will consist of measurement of antigen-specific antibody in serum and cervico-vaginal secretions as these techniques are well established in the literature. The other assays will be exploratory and methodological development.

Specifically we will characterise:

1. Concentration, neutralising activity and isotype profile of antigen-specific antibody in serum and cervico-vaginal secretions

    Using published ELISA and Luminex[11, 12] assays with L1 proteins as coating antigens, and virus neutralisation assay, we will measure the levels of IgG and IgA L1-specific antibody in serum and mucosal secretions. A 'mucosal' response will be characterised by IgA>IgG, and a systemic/nasal response by the reverse.

2. Frequency and isotype profile of antigen-specific antibody secreting cells in blood

    We will adapt published ELISpot assays[3, 14-16] (to detect single cells secreting antigen specific antibody *in vitro*) we have developed to detect responses to nasal or oral administration of antigen, to employ L1 proteins as coating antigens. By enumerating IgG and IgA L1-specific antibody secreting cells trafficking in the blood a 'mucosal' response will be characterised by IgA>IgG, and a systemic/nasal response by the reverse. Expression of mucosa-associated homing markers on antigen-specific B cells in blood can be measured by bead extraction, but this is not planned in this study.

3. Frequency and profile of regulatory, memory and mucosa-associated homing marker expression on antigen-specific T cells in blood measured by CFSE proliferation assay

    We will update our published T cell assays[1] to detect antigen specific T cells trafficking in the blood following challenge responding *in vitro* to antigen stimulation, using the flow cytometry based CFSE proliferation assay running on a Guava flow cytometer. In addition to enumerating numbers of T cells we will characterise intracellular cytokine expression and cell surface markers of responding cells including β7 integrin, CCR10, L-selectin and other markers associated with mucosal or systemic homing[6], and markers of memory (CD45RO/RA) and regulatory function (Foxp3).

4. Profile of cytokine secretion by peripheral blood mononuclear cells in response to *in vitro*

antigen stimulation measured by ELISA By measuring secretion of cytokines into the supernatant of cultures of PBMCs stimulated with L1 antigens we will be able to determine mucosal patterns of response (IL17) and the Th1: Th2 bias.

2.4 Preclinical and clinical data

Please insert a summary of findings from non-clinical studies that potentially have clinical significance and from clinical studies relevant to the study (please reference all published findings)

This study builds on and extends a series of published studies we have undertaken using licensed vaccines as safe human challenge agents to induce an immune response that can then be characterised. Using this approach we have characterised the profile of B and T cell responses following oral and nasal administration and intramuscular injection of model antigens[1-4].

Extensive preclinical and clinical data in support of the suitability of the antigens in the Gardasil preparation as challenge agents, and their safety evaluation, are presented in detail in section 5 of the Gardasil SPC (Appendix 3)

Gardasil is an adjuvanted non-infectious recombinant quadrivalent vaccine prepared from the highly purified virus-like particles (VLPs) of the major capsid L1 protein of HPV types 6, 11, 16 and 18. The VLPs contain no viral DNA, they cannot infect cells, reproduce or cause disease. HPV only infects humans, but animal studies with analogous papillomaviruses suggest that the efficacy of LI VLP vaccines is mediated by the development of a humoral immune response.

In clinical studies, 99.9%, 99.8%, 99.8%, and 99.6% of individuals who received Gardasil became anti-HPV 6, anti-HPV 11, anti-HPV 16, and anti-HPV 18-seropositive, respectively, by 1 month Postdose 3 across all age groups tested. Gardasil induced high anti-HPV Geometric Mean Titres (GMTs) 1 month Postdose 3 in all age groups tested.

The efficacy for HPV 16/18 related CIN 2/3 or AIS is based on data from protocols 005 (16-related endpoints only), 007, 013, and 015. The efficacy for all other endpoints is based on protocols 007, 013, and 015. Results of individual studies support the results from the combined analysis. Gardasil was efficacious against HPV disease caused by each of the four vaccine HPV type.

2.5 Population

Please insert a description of the population to be studied (including sample size, participant group, patient group (if applicable)). Please indicate if a vulnerable population is to be studied

- We will recruit a total of 18 healthy female volunteers, aged 18-35.
- All subjects will be healthy, and will provide written informed consent.
- No vulnerable groups will be included.
- From previous experience we expect the median age to be around 23 years old.
- We expect around half the subjects to be naive for the challenge agents[12], while the remainder will have had some exposure to one or more of the antigens.

As group size is small in this hypothesis generating study we have elected to recruit a narrow age range, to avoid possible bias due to age-related (hormonal) changes in immune response, especially in the genital tract.

Group 1:

We will initially recruit SIX subjects into a positive response group that will receive INTRAMUSCULAR challenge. This will enable us to generate positive reference samples and develop the novel assays, as it is known that intramuscular immunisation with L1 antigens induces systemic and mucosal immune responses.[11]

Group 2:
Once the novel assays are performing satisfactorily, we will recruit a further TWELVE subjects into the group to receive SUBLINGUAL challenge. It may not be necessary for subjects in group 1 to have completed the protocol before group 2 is recruited.

2.6 Dose Rationale
Please insert a description of, and justification for the, route of administration, dosage, dosage regimen and treatment period(s)

A single 0.5 mL vial of "Gardasil" vaccine has been shown through extensive clinical trials to be the optimum safe and immunogenic dose for intramuscular immunisation, and is licensed for use in healthy adult women the UK.

To enable this scientific study to complete within the time and resources available, a shortened 0, 1, 4 month challenge regimen will be employed, which is based on the recommendation of the Gardasil SPC section 4.2: "*If an alternate vaccination schedule is necessary, the second dose should be administered at least one month after the first dose and the third dose should be administered at least 3 months after the second dose*".

Although sublingual application of antigen is likely to be less efficient at stimulating immune responses due to the physical barrier to passive antigen entry across the sublingual mucosa, the extensive human safety profile of this dose level and dose regimen when injected makes it an appropriate choice for this initial hypothesis generating study.

The tiny (microgram) amounts of L1 protein and alum present no credible risk if swallowed, when compared with the huge (gram) amounts of protein (and other particulates such as toothpaste) swallowed daily. Any swallowed L1 or alum is expected to be immediately degraded by gastric acid and digestive enzymes.

2.7 Risk/Benefits

Please insert a summary of the known and potential risks & benefits, if any, to human subjects

2.7.1 Potential Risks

2.7.1.1 General risks

Gardasil does not contain thiomersal preservative. It does not contain live organisms and cannot cause HPV infection or disease. The VLPs contain no viral DNA, they cannot infect cells, reproduce, or cause disease.

Venepuncture may be associated with bruising, localised discomfort and fainting. Dedicated clinical facilities, including phlebotomy chairs will be used and staff must be trained and experienced in venepuncture. The blood volumes drawn over 20 weeks do not present a significant risk of causing anaemia in a healthy person, and subjects must be screened for anaemia before entry.

Vaginal speculum examination can be uncomfortable, and staff must be experienced in the technique. Collection of secretions onto single-use, sterile, soft ophthalmic sponges (developed to collect tears) is painless and risk-free. A dedicated room complete with colposcopy couch, private changing and showering area, must be used to minimise subject discomfort and ensure privacy.

Clinical examination and screening blood tests may disclose medical conditions such as HIV, sexually transmitted disease, cervical cancer. Staff taking consent must be trained in pre-test discussions and the Principal Investigator is an Infectious Diseases clinician who can inform subjects of the diagnoses, answer questions or concerns, and arrange appropriate follow-up with the subject's consent.

2.7.1.2 Intramuscular injection

The following text is taken from sections 4.8 and 4.9 of the Gardasil SPC (Appendix 3):

The following vaccine-related adverse reactions were observed among recipients of Gardasil at a frequency of at least 1.0% and also at a greater frequency than observed among placebo recipients. They are ranked under headings of frequency using the following convention: [Very Common (1/10); Common (1/100, <1/10); Uncommon (1/1,000, <1/100); Rare (1/10,000, <1/1,000); Very Rare (<1/10,000), including isolated reports]
General disorders and administration site conditions:
Very common: pyrexia.
Very common: at the injection site: erythema, pain, swelling.
Common: at the injection site: bruising, pruritus.
In addition, in clinical trials adverse reactions that were judged to be vaccine- or placebo-related by the study investigator were observed at frequencies lower than 1%:
Respiratory, thoracic and mediastinal disorders:
Very rare: bronchospasm.
Skin and subcutaneous tissue disorder:
Rare: urticaria.
Seven cases (0.06%) of urticaria were reported in the Gardasil group and 17 cases (0.18%) were seen in the adjuvant-containing placebo group.
In the clinical studies, subjects in the Safety Population reported any new medical conditions during the follow-up of up to 4 years. Among 11,778 subjects who received Gardasil and 9,686 subjects who received placebo, there were 26 cases of non-specific arthritis/arthropathy reported, 19 in the Gardasil group and 7 in the placebo group.

Post Marketing Experience
Post Marketing adverse events have been spontaneously reported for Gardasil and are not listed above.

Because these events were reported voluntarily from a population of uncertain size, it is not possible to reliably estimate their frequency or to establish, for all events, a causal relationship to vaccine exposure.
Blood and lymphatic system disorders: lymphadenopathy.
Immune system disorders: hypersensitivity reactions including anaphylactic/anaphylactoid reactions.
Nervous system disorders: Guillain-Barré syndrome, dizziness, headache, syncope.
Gastrointestinal disorders: nausea, vomiting.
Musculoskeletal and connective tissue disorders: arthralgia, myalgia.
General disorder and administration site conditions: asthenia, fatigue, malaise

Overdose
There have been reports of administration of higher than recommended doses of Gardasil. In general, the adverse event profile reported with overdose was comparable to recommended single doses of Gardasil.

Anaphylaxis
Staff administering challenge agents must be trained in resuscitation and management of anaphylaxis, with regular refreshers. The clinical site will maintain a crash trolley, oxygen, defibrillator and SOPs to manage acute medical emergencies associated with immunisation, including access to emergency services. Adequate numbers of trained staff must be present during challenges.

2.7.1.3 Topical sublingual application
The risk profile associated with topical sublingual application has not be specifically evaluated, but is expected to be considerably less than after injection given the low level of protein expected to passively cross the oral mucosa, compared with the dose delivered intramuscularly. Single-dose and repeated-dose toxicity and local tolerance studies revealed no special hazards to humans (Gardasil SPC section 5.3 Appendix 3). Natural infection with HPV (which can also be oral[13]) is initially asymptomatic and not associated with localised reactions to L1 proteins on the viral surface that contact the mucosal surface (warts develop after 3 weeks due to the effects on cell growth of internal viral proteins and cancer-promoting genes - not present in the Gardasil preparation).

The tiny (microgram) amounts of L1 protein and alum present no credible risk if swallowed, when compared with the huge (gram) amounts of protein (and other particulates such as toothpaste) swallowed daily. Any swallowed L1 or alum is expected to be immediately degraded by gastric acid and digestive enzymes.

As daily sublingual application of allergens over many months has been shown to be required to induce tolerance to allergic responses, no tolerising effect is expected from the three application of these virus-derived protein antigens at 0, 1 and 4 months proposed here.

2.7.2 Potential Benefits
By better understanding the common mucosal immune system we will learn more about how the body handles pathogen antigens presented via systemic or mucosa-associated lymph nodes. This may advance our understanding of immune responses to viral infections, and inform the design of mucosal vaccine delivery systems. We will also develop novel assays, and a model of mucosal immune challenge with model viral antigens.

Each subject screened will receive a free basic medical health check.

2.7.2.1 Intramuscular injection
Section 5.1 of the Gardasil SPC (Appendix 3) details the level of benefit associated with intramuscular immunisation.

In clinical studies, 99.9%, 99.8%, 99.8%, and 99.6% of individuals who received Gardasil became anti-HPV 6, anti-HPV 11, anti-HPV 16, and anti-HPV 18-seropositive, respectively,

by 1 month Post-dose 3 across all age groups tested. Gardasil induced high anti-HPV Geometric Mean Titres (GMTs) 1 month Post-dose 3 in all age groups tested. This level of immunity was associated with high levels (100%) of protection against cervical cancer lesions. Subjects over 18 are not eligible for the free NHS mass immunisation programme and so will benefit from a free vaccination (currently approximately £120 per injection).

Subjects already infected with HPV can expect to gain no benefit against the serotype with which they are infected, but will be protected against other serotypes. Although we will use an alternative schedule of immunisation, it is one recommended by the Gardasil SPC and subjects can expect to receive a high level of benefit in protection against HPV infection and associated cervical cancer or genital warts.

2.7.2.2 Topical sublingual administration

No data is available for the level of immunity induced by sublingual immunisation. However, due to physical barriers limiting antigen penetration across the oral mucosa, and destruction of swallowed antigen by gastric acid and digestive enzymes, it is expected that any immune responses induced will be low level and non-protective.

It is theoretically possible that topical sublingual exposure may prime subjects for an enhanced immune response to subsequent intramuscular injection, however this is speculative. Subjects should therefore not expect to gain any benefit from inclusion in this arm of the study.

2.8 Subject population(s) for analysis

Please insert the number of subjects planned to be enrolled. In multi-centre studies, the number of enrolled subjects projected for each study site should be specified. Provide a reason for choice of sample size, including reflection on (or calculations of) the power of the study and clinical justification

All subjects will be recruited at one site.

We will recruit a total of 18 healthy female volunteers, aged 18-35.

From previous experience we expect the median age to be around 23 years old.

We expect around half the subjects to be naive for the challenge agents[12], while the remainder will have had some exposure to one or more of the antigens.

We will initially recruit the first SIX subjects into a group that will receive INTRAMUSCULAR challenge.

We will subsequently recruit a further TWELVE subjects into the group to receive SUBLINGUAL challenge.

The numbers have been selected according to our previous experience with mucosal antigen delivery, and the expected circa 100% response rate seen with intramuscular immunisation with HPV vaccines[17]. As this is a hypothesis generating study it is not powered to enable between-group subgroup analysis. A response after challenge in any of the assays will constitute a valid response to challenge. In addition to providing data on antigen handling via systemic or mucosal routes, this study will generate data on the feasibility of sublingual challenge studies, and guide the choice of suitable assays to characterise immune responses in subsequent larger studies.

2.9 Study design/type

Please insert a description of the type/design of the study to be conducted (e.g. double-blind placebo controlled, parallel design) and a schematic diagram of the study design, all procedures and stages.

- **Physiology study using a challenge agent, not a CTIMP**
- **Open label, non-randomised, hypothesis generating study**

2.9.1 Schedule of visits and procedures

Visit no.	1	2	3	4	5	6	7	8	9
Study day no.	-28 to -2	0	7	28	35	56	112	119	140
Study week no.		0	1	4	5	8	16	17	20
Study month no.		0		1		2	4		5
Informed consent	X								
In/exclusion criteria	X	X		X			X		
Demography	X								
Pulse, temperature	X	X		X			X		
Heart (including blood pressure), lungs, abdomen, oral cavity; cervical speculum	X								
Pregnancy test									
Blood	X								
Urine		X		X			X		
Urinalysis (dipstick)	X								
Blood for exclusions (15 mL): FBC, HBV sAg, HIV1&2 Ab, HCV Ab*	X								
**Challenge**									
Group 1: 0.5 mL Gardasil IM		X		X			X		
Group 2: 0.5 mL Gardasil sublingual		X		X			X		
Blood for B / T cell analysis (20-50 mL)†		BT	B	TB	B	TB	BT	B	TB
Blood for serum antibody (5 mL)		X	X	X	X	X	X	X	X
Cervico-vaginal secretions for antibody		X		X		X	X		X
Volume of blood drawn (Total: 380 mL)	15	55	25	55	25	55	55	25	55

** FBC: Full Blood Count. HBV sAg: Hepatitis B virus surface antigen. HIV1&2 Ab: Antibody for HIV1 & 2 viruses. HCV Ab: Antibody to Hepatitis C virus*

† 50 mL total split as 15 mL to B cell assay / 35 mL to T cell assay on days when both taken. Otherwise 20 mL for B cell assays and 50 mL for T cell assays on days when taken alone

2.9.2 Acceptable Visit Windows

Visits within the following window periods will not constitute a protocol deviation:
Visits 3, 5 and 8: +/- 1 day
Visits 4, 6, 7 and 9: +/- 2 days

Given the exploratory nature of this study, it will be acceptable to collect and analyze samples taken at the *closest possible time* to the scheduled visit, if the subject is unable to make the scheduled visit. No additional visits are allowed.

2.9.3 Details of study visits

The length of visits will vary according to the purpose of each particular visit.

After initial contact expressing interest in the study, subjects will receive verbal information and an Information Sheet which they are encouraged to discuss this with their GP, friends and family. After at least 24 hours subjects will be eligible to sign written consent, having had a pre-test discussion relating to HIV testing. They are then eligible to make Visit 1.

Visit 1: Eligibility check
(Between 28 and 2 days before visit 2)
Basic demographic information will be collected to identify subject, and to check inclusion/exclusion criteria. No information relating to race, height, weight will be collected. Past and current medical and medication history will be recorded to check exclusion criteria. A general medical examination (heart, lungs, abdomen, mouth, vagina-cervix (with speculum) will be performed. Pulse and temperature recorded.

Blood will be taken and tested for Full Blood Count, HIV and Hepatitis B and C infections, pregnancy. Urine dipstick analysis.

Visits 2, 4 and 7: Antigen delivery and immunology response follow-up
(Day 0, 28 and 112)
Pulse and temperature recorded. General state of health, concomitant medications elicited to determine any exclusion/withdrawal criteria. Urine pregnancy test carried out.
Blood taken for immunology assays:

- Serum on all visits
- B cell assays visits 2 & 7
- T cell assays on all visits

Vaginal-cervical secretions collected onto a soft ophthalmic sponge using a speculum, for immunology assays.

The HPV antigens are given sublingually (as drops placed onto the undersurface of the tongue) or by injection (into the upper arm muscle) - according to study group.

Visits 3, 5, 6 and 8: Immunology response follow-up
(Day 7, 35, 56, 119)
Blood taken for immunology assays:

- Serum on all visits
- B cell assays visits 3, 5 & 8
- T cell assays visit 6

Vaginal-cervical secretions collected onto a soft ophthalmic sponge using a speculum at visit 6, for immunology assays

Visit 9: Final immunology response follow-up
(Day 140)
At the final visit blood will be taken for T cell and serum immunology assays. Vaginal-cervical secretions collected onto a soft ophthalmic sponge using a speculum, for immunology assays.

On ***one only*** of visits 2, 4, 6, 7 ***or*** 9 subjects will be invited to collect a vaginal sample using the self-inserted and self-removed ‘Instead cup’ in addition to weck-cel samples. Participation in this procedure is voluntary and not required for protocol compliance. This sample may be used for immunology assays or by collaborators investigating the *in vitro* effects of healthy cervico-vaginal samples on vaccine antigen integrity. Samples will be processed according to laboratory SOPs of the collaborating laboratories.

This concludes a subject's participation.

**2.10 Data Identification**<br>*Please insert the identification of any data to be recorded directly on the Case Report Forms (CRFs) (i.e. no prior written or electronic record of data), and to be considered to be source data*
Source data will consist of the subject demographics, results of screening exclusion assays, physical examination, administration of challenge agents, details of vials used, allocation to group, and will be entered directly onto the CRFs.<br><br>Results of immunological assays will be entered onto paper worksheets, which will be considered source data. Electronic data will be used for subsequent analysis.

**2.11 Primary and/or Secondary Study Endpoints**<br>*Please insert a specific statement of the primary endpoints and the secondary endpoints, if any, to be measured during the study*
No primary and secondary endpoints are defined in this hypothesis-generating study.<br><br>A number of exploratory variables will be measured:<br>1. Concentration, neutralising activity and isotype profile of antigen-specific antibody in serum and cervico-vaginal secretions measured by ELISA and/or LUMINEX assay and virus neutralisation assay<br>2. Frequency and isotype profile of antigen-specific antibody secreting cells in blood measured by ELISPOT assay<br>3. Frequency and expression profile of mucosa-associated homing, memory and regulatory markers on antigen-specific T cells in blood in response to *in vitro* antigen stimulation measured by Flow Cytometry (FACS) and CFSE proliferation assay<br>4. Profile of cytokine secretion by peripheral blood mononuclear cells in response to *in vitro* antigen stimulation measured by ELISA

**2.12 Randomisation**<br>*Please insert a description of the measures taken to minimize/avoid bias, including randomisation (please include a description of the method) and blinding (if applicable)*
There will be no randomisation.<br><br>The nature of the challenge makes blinding impossible and no placebo or comparator will be used. Each subject acts as their own control, comparing pre-challenge with post-challenge time points.<br><br>The first 6 subjects will be recruited into the intramuscular challenge group, to allow assays to be developed and positive control samples generated, as this route of challenge is known to reliably induce mucosal and systemic immune responses.<br><br>The remaining 12 subjects will then be entered into the sublingual group.

**2.13 Maintenance of randomisation codes**<br>*Please insert a description of the maintenance of the randomisation codes and the procedure for breaking codes*
Not applicable.

2.14 Inclusion criteria

Please insert subject inclusion criteria

All subjects must satisfy the following criteria at study entry:

1. A female adult volunteer aged between 18 and 35 years old.
2. Subjects who the investigator believes can and will comply with the requirements of the protocol.
3. Provide written informed consent following a detailed written explanation of participation in the protocol.
4. They are in good health as determined by medical history, physical examination, haematology testing, and clinical judgement before entering into the study.
5. They are available for the whole duration of the study.
6. If of childbearing potential, must have a negative pregnancy test before each immunisation.
7. They have not donated blood during 3 months prior to study entry and agree to not donate for 3 months after the end of their participation in the study.
8. They are eligible for free medical treatment

2.15 Exclusion criteria

Please insert subject exclusion criteria

Subjects will be considered ineligible to enter the study if they present a condition which *could interfere with normal immune responses* to the challenge agent, including if they meet any of the following exclusion criteria:

1. They have already been vaccinated with an HPV vaccine
2. They have participated in a clinical trial in the last 6 months in which they have been exposed to an investigational product (pharmaceutical product or placebo or device) or concurrent participation in another clinical research study at the time of enrolment.
3. Use of any investigational or non-registered product (drug or vaccine) within 30 days preceding the first dose of challenge agent, or planned use during the study period.
4. They are pregnant or breast-feeding.
5. They have a known or suspected ongoing cervico-vaginal disease, malignancy or abnormality discovered at time of screening.
6. They present in the samples obtained at the screening visit: positive results for HIV, HBs Ag, anti-HBc and anti-HCV antibody, a clinically significant abnormality in haematology. Normal ranges will be defined by the pathology laboratory undertaking assays.
7. They have a clinically significant acute or chronic pulmonary, cardiovascular, hepatic or renal functional abnormality, blood or neurological disorders, immune dysfunction, autoimmune diseases, diabetes (excluding history of gestational diabetes), or malignancy at the time of enrolment, as determined by medical history, physical examination or laboratory screening tests.
8. They have received any form of immunosuppressive therapy in the past 6 months.
9. They are receiving any medications via vaginal route (as this may interfere with collection of samples).
10. They have any tongue or frenulum piercings or oral jewellery that may interfere with

sublingual delivery.

11. They have received blood products or immunoglobulins 120 days prior to enrolment.
12. They have thrombocytopaenia or any coagulation disorder (because bleeding may occur following an intramuscular administration in these individuals).
13. Any other medical, psychiatric or social condition, drug treatment, occupational or other responsibility that, in the judgement of the investigator, would interfere with or serve as a contradiction to adherence to the study protocol or ability to give informed consent.
14. Individuals who cannot read or speak fluent English.

2.16 Subject withdrawal criteria

Please insert subject withdrawal criteria (i.e. terminating study treatment) and procedures specifying:
(a) When and how to withdraw subjects from the study.
(b)The type and timing of the data to be collected for withdrawal of subjects.
(c)Whether and how subjects are to be replaced.
(d)The follow-up for subjects withdrawn from study treatment.

(a) When and how to withdraw subjects from the study. (b) The type and timing of the data to be collected for withdrawal of subjects.

According to the Gardasil SPC (Appendix 3):

(i) "*Individuals who develop symptoms indicative of* ***hypersensitivity*** *after receiving a dose of Gardasil should not receive further doses of Gardasil*." - These subjects will be withdrawn from the study at that point. No further data will be collected.

(ii) "*Administration of Gardasil should be postponed in subjects suffering from an* ***acute severe febrile illness***." - Such subjects will not be withdrawn but challenges will be postponed until the subjects fully recover. This will be recorded as a protocol deviation and subsequent timepoints will be calculated relative to the date of the postponed immunisation.

(iii) In addition to the above, subjects experiencing a **suspected drug reaction**, *including* those listed on the Gardasil SPC, will be withdrawn if in the opinion of the Principal Investigator the severity of the reaction may interfere with the study objectives, or if there is a possibility that the reaction may re-occur on subsequent challenge. No further data will be collected.

(iv) Any subject that becomes **pregnant**, or who tests positive in a pregnancy test taken during the protocol, will be immediately withdrawn. The subject will not undertake any further investigations, sample collections or challenges. The subject will be followed up and the outcome of the pregnancy recorded in the source notes.

(v) If new information becomes available that unfavourably alters the risk-benefit analysis of Gardasil, the Principal Investigator may halt the study and withdraw all subjects that require further challenges, as this is a hypothesis generating study and subjects will not be exposed to increased risk from premature discontinuation. The Principal Investigator will discuss the situation with subjects in the group receiving intramuscular immunisation, to determine whether they would benefit from continuing any incomplete schedule via their GP or travel clinic. However no further Gardasil challenges will be given by clinical staff. Subjects who have completed all scheduled challenges will not be withdrawn.

(vi) "*However, the presence of a* ***minor infection****, such as a mild upper respiratory tract infection or low-grade fever, is not a contraindication for immunisation*." Subjects *will* be challenged according to the expected study schedule in this situation. A note of the intercurrent illness will be made in the source notes.

(c) Whether and how subjects are to be replaced.

There will be no replacement of withdrawn subjects, as this hypothesis generating study is not powered to detect any specific frequency of events.

(d) The follow-up for subjects withdrawn from study treatment.

Subjects withdrawn due to an adverse drug reaction will be followed-up according to the ▼Yellow Card scheme.

There will be no follow-up of other withdrawn subjects.

2.17 Study treatment

Please insert a description of the study treatment(s) and the dosage and dosage regimen. Also include a description of the dosage form, packaging, and labelling. Please indicate storage conditions and outline regulatory requirements (where applicable)

Name of challenge agents
Gardasil, suspension for injection in a pre-filled syringe. Human Papillomavirus Vaccine [Types 6, 11, 16, 18] (Recombinant, adsorbed).

Qualitative and quantitative composition
1 dose (0.5 ml) contains approximately:
Human Papillomavirus Type 6 L1 protein 20 micrograms
Human Papillomavirus Type 11 L1 protein 40 micrograms
Human Papillomavirus Type 16 L1 protein 40 micrograms
Human Papillomavirus Type 18 L1 protein 20 micrograms

L1 protein in the form of virus-like particles produced in yeast cells (*Saccharomyces cerevisiae* CANADE 3C-5 (Strain 1895)) by recombinant DNA technology. Adsorbed on amorphous aluminium hydroxyphosphate sulphate adjuvant (225 micrograms Al)

Dosage regimen
Three challenges with 0.5 mL of Gardasil will be given either sublingually or intramuscularly on months 0, 1 and 4 (as recommended in the Gardasil SPC).

Formulation
Suspension for injection in a pre-filled syringe. Prior to agitation, Gardasil may appear as a clear liquid with a white precipitate. After thorough agitation, it is a white, cloudy liquid.

Excipients
Sodium chloride L-histidine Polysorbate 80 Sodium borate Water for injections.

Incompatibilities
In the absence of compatibility studies, Gardasil must not be mixed with other medicinal products.

Shelf life
3 years.

Special precautions for storage
Store in a refrigerator (2°C - 8°C). Do not freeze. Keep the pre-filled syringe in the outer carton in order to protect from light.

Nature and contents of container
0.5 ml suspension in a pre-filled syringe (Type 1 glass) with plunger stopper (siliconized FluroTec-coated bromobutyl elastomer or non- coated chlorobutyl elastomer) and tip cap (bromobutyl) without needle.

Regulatory requirements

Review of this protocol by MHRA confirmed that it is not a Clinical Trial of an Investigational Medicinal Product (IMP) as defined by the EU Directive 2001/20/EC. There is no requirement to submit a Clinical Trial Authorisation (CTA) to the MHRA.

Gardasil will be purchased from an approved supplier, and although this is not a clinical trial it will be used under the general direction and supervision of the Principal Investigator who is a registered medical practitioner.

Standard reporting of adverse drug reactions to MHRA will be conducted under the ▼Yellow Card Scheme.

2.18 Duration
Please insert the expected duration of subject participation and a description of the sequence and duration of all study periods, including follow-up, if any

Subjects will participate for up to 6 months including: a 1 maximum month screening period, followed by a 5 month follow-up period after the first immunological challenge.

2.19 Treatment of subjects
Please insert the treatment(s) to be administered, including the name(s) of all the product(s), the dose(s), the dosing schedule(s), the route/mode(s) of administration, and the treatment period(s), including follow-up period(s) for subjects for each treatment/study treatment group/arm of the study

Although this is not a clinical trial, the Principal Investigator shall maintain a Delegations Log of study staff authorised to administer challenge agents, who will have the appropriate training in immunisation and resuscitation techniques (in compliance with section 3 "Characteristics of staff" of the Department of Health "Patient Group Direction (PGD) for the supply and administration of Human Papillomavirus Vaccine" [Appendix 2]) which will be recorded on an up to date CV and training record held in the site file.

A single 0.5 mL dose of Gardasil HPV vaccine will be used as an immunological challenge in all subjects in both groups.

All subjects in both groups will receive a challenge on month 0, 1 and 4.

Group 1: Six subjects will receive the challenge via intramuscular injection into the deltoid muscle (on the contralateral side to their dominant hand), following the instructions in section 6.6 of the Gardasil SPC (Appendix 3).

Group 2: Twelve subjects will receive the challenge via drops applied topically to the sublingual surface of the tongue according to a predefined SOP (Appendix 1)

2.20 Discontinuation
Please insert a description of the stopping rules or discontinuation criteria for individual subjects, parts of the study, and entire study. Please include any information on dose modification procedures

Individual subject stopping/discontinuation is detailed in section 2.16

The study will be stopped if a suspected adverse drug reaction that is not listed on the "Gardasil" SPC is reported to the MHRA using the ▼Yellow Card scheme. In this case no further challenges will be administered and subjects already entered will be withdrawn from immunological follow-up at the discretion of the Principal Investigator.

If new information becomes available that unfavourably alters the risk-benefit analysis of Gardasil, the Principal Investigator may halt the study and withdraw all subjects that require further challenges, as this is a hypothesis generating study and subjects will not be exposed to increased risk from premature discontinuation. The Principal Investigator will discuss the situation with subjects in the group receiving intramuscular immunisation, to determine whether they would benefit from continuing any incomplete schedule via their GP or travel clinic. However no further Gardasil challenges will be given by clinical staff. Subjects who have completed all scheduled challenges will not be withdrawn, and will complete all scheduled follow-up visits. No new subjects will be recruited.

2.21 Challenge Agents Accountability
Please insert accountability procedures including the placebo(s) and comparator(s), if any

Vaccine Institute GCP-compliant SOPs will be used to record receipt, administration, loss, and destruction of unused vials of Gardasil.

2.22 Accountability procedure
Please insert the procedure for accounting for missing, unused and spurious data

The SOPs and laboratory worksheets of the Vaccine Institute will be used to record and monitor data.

2.23 Monitoring subject compliance
Please insert the procedures for monitoring subject compliance

All challenges will be administered directly by trained and delegated study staff.

2.24 Permitted medication
Please insert medication(s)/treatment(s) permitted (including rescue medication) and not permitted before and/or during the study (e.g. concomitant medicines)

Subjects will be withdrawn at the point that they commence any medications or transfusions referred to in the Exclusion Criteria.

All other concomitant medications are permitted.

See also section 4.5 of Appendix 3 Gardasil SPC "Interaction with other medicinal products and other forms of interaction"

2.25 Efficacy parameters
Please insert the specifications of the efficacy parameters

Not a CTIMP: no efficacy parameters will be measured.

2.26 Method and timing for efficacy parameters
Please insert methods and timing for assessing, recording and analyzing efficacy parameters

Not applicable.

2.27 Safety parameters
Please insert specifications for safety parameters

Not a CTIMP: no safety parameters will be measured.

2.28 Adverse event reporting
Please insert details of expected adverse events and the procedures for eliciting reports of and for recording and reporting of these adverse events and intercurrent illnesses.

As the challenge agents are not IMPs there is no specific reporting of adverse events or SUSARS.

Suspected adverse drug reactions will be reported to MHRA using the s ▼Yellow Card

scheme.

**2.29 Adverse event follow-up** *Please insert the type and duration of the follow-up of subjects after adverse events*
Suspected adverse drug reactions will be followed to resolution or stabilisation and reported to MHRA using the ▼Yellow Card scheme.

**2.30 Statistical methods** *Please insert a description of the statistical methods to be employed including timing of any planned interim analysis(ses), level of significance to be used*
No primary and secondary endpoints are defined in this hypothesis-generating study.<br>A number of exploratory variables will be measured. As this is a hypothesis generating study it is not powered to enable between-group subgroup analysis. A response after challenge in any of the assays will constitute a valid response to challenge.<br>Appropriate statistical tests of significance (parametric or non-parametric) will be used for between-group, or between-time-point analyses of exploratory variables, according to the nature and size of the dataset, and in consultation with a statistician before publication.

**2.31 Subject analysis** *Please insert the selection of subjects to be included in the analyses (e.g. all randomised subjects, all dosed subjects, all eligible subjects, evaluable subjects)*
Data will be analysed for all subjects receiving at least one challenge.

**2.32 Method and timing for safety parameters** *Please insert methods and timing for assessing, recording and analyzing safety parameters*
Not applicable.

**2.33 Termination criteria** *Please insert the criteria for the termination of the study*
The study will be terminated if:<br>1. Insufficient subjects have been recruited within 12 months of administering the first challenge to the first subject. In this case all subjects entered will complete the protocol.<br>2. A suspected adverse drug reaction that is not listed on the "Gardasil" SPC is reported to the MHRA using the ▼Yellow Card scheme. In this case no further challenges will be administered and subjects already entered will be withdrawn from immunological follow-up at the discretion of the Principal Investigator.

The study conduct shall comply with all relevant laws of the EU if directly applicable or of direct effect and all relevant laws and statutes of the UK country in which the study site is located including but not limited to, the Human Rights Act 1998, the Data Protection Act 1998, the Medicines Act 1968, and with all relevant guidance relating to medicines and clinical studies from time to time in force including, but not limited to, the ICH GCP, the World Medical Association Declaration of Helsinki entitled 'Ethical Principles for Medical Research Involving Human Subjects' (2008 version).

This study will be conducted in compliance with the protocol approved by the REC and according to GCP standards. No deviation from the protocol will be implemented without the prior review and approval of the sponsor and REC except where it may be necessary to eliminate an immediate hazard to a research subject. In such case, the deviation will be reported to the sponsor and REC as soon as possible.

**2.34 Ethical considerations**<br>*Please insert description of ethical considerations relating to the study*
The informed consent shall explicitly make reference to the possibility that an abnormality may be incidentally discovered such as a haematological disorder, immunological deficiency, cervico-vaginal disease or malignancy, chronic viral infection, etc.<br><br>Subjects must be eligible for free medical treatment as part of the inclusion criteria. Any medical conditions discovered at the time of screening may therefore be managed at no cost to the subject.<br><br>Subjects will be fully informed of any abnormal conditions discovered by screening tests, the physical examination, or the immunology assays (e.g. an immunoglobulin or lymphocyte deficiency) and the consequences and necessary management explained by the Principal Investigator who is a Consultant Physician. The Principal Investigator will seek the permission of the subject to communicate the findings to their General Practitioner for further action, and ensure that the communication has been received. Subjects unwilling to have details of any condition reported to their GP will be encouraged to reconsider, but their wishes will be respected.<br><br>Subjects will be tested for HIV, hepatitis B and C at screening. This is required as HIV or chronic hepatitis virus infections will considerably affect the immune response to the challenge agents and skew the data. Clinical samples from infected subjects also pose a risk to laboratory staff as several of the exploratory assays cannot be run under Universal Precautions. Subjects will undergo a pre-test discussion by a qualified GUM nurse experienced in HIV and HIV-related issues, as part of the informed consent process. Any positive results will be given to the subject in person by the Principal Investigator who is a Consultant Infectious Diseases/HIV Physician, and who will personally ensure that necessary follow-up and management is arranged if the subject gives their permission. Subjects unwilling to have details reported to their GP will be encouraged to attend a GUM clinic where anonymity and confidentiality may be assured, but their wishes will be respected.<br><br>Subjects must consent to allow study staff to inform their General Practitioner of their participation in the study, nature and number of challenge agents administered, and to seek information that may contraindicate their safe exposure to the challenge agents.<br><br>Subjects entering the sublingual administration group will need to be fully aware, through written information in the informed consent sheet reinforced by verbal communication from study staff, that although they will have been exposed to the Gardasil vaccine they cannot expect to have protective immunity, and should consider themselves unimmunised in the event of being offered HPV immunisation in the future. Although specific trials have not been conducted, it is current practice that boosting with any of the available HPV vaccines is acceptable.

**2.35 QC & QA**<br>*Please insert details of procedures for generation, recording and reporting of study data in compliance with the protocol, GCP standards and any other applicable regulatory requirements*
Results of screening pathology tests will be generated by TDL on printed sheets which will be initialled and dated by the Principal Investigator and filed in the Source Notes.<br>Any clinically significant result will be marked "CS", and non-clinically significant results "NCS" by a trial Physician on the original test result printout. All annotations will be initialled

and dated by a trial Physician.

Results of immunology assays will be recorded and checked by technicians undertaking the tests and signed, and dated by them on paper worksheets.

Study staff will be responsible for data entry and verification for those data they enter into the study documentation.

All data, both electronic and paper, will be held in secure (locked / password protected as appropriate) facilities within the Vaccine Institute under the provisions of the SGUL Data Protection Act registration and local governance requirements.

2.36 Deviation reporting

Please insert the procedures for reporting any deviation(s) from the original statistical plan. Any deviation(s) from the original statistical plan should be described and justified in the protocol and/or in the final report as appropriate

Not applicable.

2.37 Subject analysis

Please insert a statement that the investigator(s) will permit study related monitoring, audits, REC review, and regulatory inspection(s), providing direct access to source data/documents*

The investigators will permit study related monitoring, audits, REC review, and regulatory inspection(s), providing direct access to source data/documents.

* Countersignature of this research protocol by the sponsor (or on behalf of the sponsor) indicates sponsor agreement with the above statement

2.38 Data storage

Please insert details of procedures for data handling, record keeping and archiving arrangements both during and post study, including location of data, accessibility rights and security provisions

All data, both electronic and paper, will be held in secure (locked / password protected as appropriate) facilities within the Vaccine Institute under the provisions of the SGUL Data Protection Act registration and local governance requirements. No archiving of data will be undertaken once the study has completed, and a study report and any publication in the literature completed.

2.39 Finance and insurance arrangements

Please insert details of source of finance for study and provide details of insurance arrangements/provider to cover the study

Finance will be provided by a grant from the European Commission Seventh Framework Programme through the EURONEUT41 consortium of which St George's is a full partner (grant reference REE0024).

Indemnity will be provided by the St George’s University of London clinical studies and trials insurance.

2.40 Publication rights

Please insert details of publication policy (e.g. ownership of data). Please note, if Trust/SGUL are sponsoring the research then the ownership rights of intellectual property lie with the institution

Publication of data will be in accordance with the provisions of the Consortium Agreement entered into between St George’s and the EURONEUT-41 Consortium funding this project.

2 Supplementary Information

2.41 Literature
Please provide reference to any literature and data that are relevant to the study and that provide background for the study

1. Castello-Branco LR, Griffin GE, Poulton TA, Dougan G, Lewis DJ. Characterization of the circulating T-cell response after oral immunization of human volunteers with cholera toxin B subunit. Vaccine 1994;12:65-72.

2. Cosgrove CA, Castello-Branco LR, Hussell T, et al. Boosting of cellular immunity against Mycobacterium tuberculosis and modulation of skin cytokine responses in healthy human volunteers by Mycobacterium bovis BCG substrain Moreau Rio de Janeiro oral vaccine. Infect Immun 2006;74:2449-52.

3. Huo Z, Sinha R, McNeela EA, et al. Induction of protective serum meningococcal bactericidal and diphtheria-neutralizing antibodies and mucosal immunoglobulin A in volunteers by nasal insufflations of the Neisseria meningitidis serogroup C polysaccharide-CRM197 conjugate vaccine mixed with chitosan. Infect Immun 2005;73:8256-65.

4. Lewis DJ, Novotny P, Dougan G, Griffin GE. The early cellular and humoral immune response to primary and booster oral immunization with cholera toxin B subunit. Eur J Immunol 1991;21:2087-94.

5. Holmgren J, Czerkinsky C. Mucosal immunity and vaccines. Nat Med 2005;11:S45-53.

6. Mestecky J, Moldoveanu Z, Russell MW. Immunologic uniqueness of the genital tract: challenge for vaccine development. Am J Reprod Immunol 2005;53:208-14.

7. Frati F, Moingeon P, Marcucci F, et al. Mucosal immunization application to allergic disease: sublingual immunotherapy. Allergy Asthma Proc 2007;28:35-9.

8. Wu HY. Induction of mucosal tolerance in SLE: a sniff or a sip away from ameliorating lupus? Clin Immunol 2009;130:111-22.

9. Song JH, Nguyen HH, Cuburu N, et al. Sublingual vaccination with influenza virus protects mice against lethal viral infection. Proc Natl Acad Sci U S A 2008;105:1644-9.

10. Cuburu N, Kweon MN, Song JH, et al. Sublingual immunization induces broad-based systemic and mucosal immune responses in mice. Vaccine 2007;25:8598-610.

11. Nardelli-Haefliger D, Wirthner D, Schiller JT, et al. Specific antibody levels at the cervix during the menstrual cycle of women vaccinated with human papillomavirus 16 virus-like particles. J Natl Cancer Inst 2003;95:1128-37.

12. Jit M, Vyse A, Borrow R, Pebody R, Soldan K, Miller E. Prevalence of human papillomavirus antibodies in young female subjects in England. Br J Cancer 2007;97:989-91.

13. D'Souza G, Agrawal Y, Halpern J, Bodison S, Gillison ML. Oral Sexual Behaviors Associated with Prevalent Oral Human Papillomavirus Infection. J Infect Dis 2009;199:1263-9.

14. Launay O, Sadorge C, Jolly N, et al. Safety and immunogenicity of SC599, an oral live attenuated Shigella dysenteriae type-1 vaccine in healthy volunteers: Results of a Phase 2, randomized, double-blind placebo-controlled trial. Vaccine 2009;27:1184-91.

15. Mills KH, Cosgrove C, McNeela EA, et al. Protective levels of diphtheria-neutralizing antibody induced in healthy volunteers by unilateral priming-boosting intranasal immunization associated with restricted ipsilateral mucosal secretory immunoglobulin a. Infect Immun 2003;71:726-32.

16. Sadorge C, Ndiaye A, Beveridge N, et al. Phase 1 clinical trial of live attenuated Shigella dysenteriae type-1 DeltaicsA Deltaent Deltafep DeltastxA:HgR oral vaccine SC599 in healthy human adult volunteers. Vaccine 2008;26:978-87.

17. Joura EA, Kjaer SK, Wheeler CM, et al. HPV antibody levels and clinical efficacy following administration of a prophylactic quadrivalent HPV vaccine. Vaccine 2008;26:6844-51.

**2.42 Supplementary details**<br>*Please insert any further relevant information to this research protocol (e.g. common toxicity criteria, tools to be used in study)*
• Appendix 1: Standard Operating Procedure for sublingual application of Gardasil challenge agent<br>• Appendix 2: Characteristics of staff delegated to administer challenge agents<br>• Appendix 3: Summary of Product Characteristics for Gardasil

Appendix 1: Standard Operating Procedure for sublingual application of Gardasil

1. Subjects must fast for 1 hour prior to challenge, but may take water freely.
2. Operator to wear gloves and plastic apron. Masks and eye protection not required.
3. One 0.5 mL pre-filled syringe of Gardasil is opened, needle not attached, and placed conveniently to hand.
4. Subjects sit in a dental chair in an upright position with the head in a level position and supported by headrest.
5. Subjects rinse the mouth with bottled water and expectorate.
6. Absorbent pads specifically designed for the purpose (e.g. “Molnlycke ‘Dry Tips’ small) applied over parotid duct openings bilaterally to absorb parotid saliva flow.
7. Tongue raised by the subject to reveal the sublingual area which is gently dried by brief application of a cotton swab without aggravating saliva flow from submandibular and sublingual glands.
8. The complete contents of the Gardasil syringe immediately (without delay to prevent saliva accumulation) dispensed to the under surface of the tongue in the area behind the sublingual fold bilaterally (white arrows on diagram below), as gentle, individual drops (not squirted) with the syringe held just above the surface and not touching the surface of the mouth. Drops to be as carefully as possible contained within the area behind the sublingual fold.

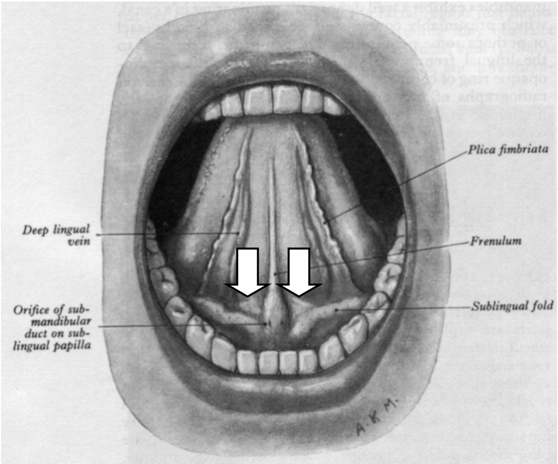

9. Subject lowers tongue and holds in gentle opposition to the floor of the mouth, trapping the contents as much as possible under the tongue. Subject to avoid swallowing. This position to be held for 15 minutes.
10. After 15 minutes has passed, cotton pads over parotid ducts removed.
11. Subject to remain in clinical site for further 30 minutes without taking anything orally.
12. Subject allowed to leave clinical site and asked to fast, including fluids, for 1 hour.

Appendix 2: Characteristics of staff delegated to administer challenge agents

(taken from Department of Health "Patient Group Direction (PGD) for the supply and administration of human papillomavirus vaccine")

Qualifications required

- Registered healthcare professional/nurse with current Nursing and Midwifery Council registration level 1 or 2.
- Assessed as competent to work with this patient group direction.

Additional requirements

- Will have undertaken training in the role, care and administration of the medicine specified in the PGD.
- Must be competent in the recognition and management of anaphylaxis.
- Must have access to a current copy of the BNF and Immunisation against infectious disease ('Green book') and comply with its recommendations (available on DH website – www.dh.gov.uk/greenbook).
- Must have access to all relevant DH advice, including the relevant CMO letters

or

- training and competent in all aspects of immunisation including contraindications and recognition and treatment of anaphylaxis.

Continued training requirements

- Annual attendance at the PCT's or workplace update on resuscitation skills and the management of anaphylaxis within the community.
- Maintenance of own level of updating with evidence of continued professional development (PREP requirements)

or

- regular updates in immunisation, vaccination, anaphylaxis and cardiopulmonary resuscitation

or

- to reinforce and update knowledge and skills in this area of practice, including basic resuscitation and anaphylaxis training, with particular reference to changes and national directives.
